# A Fishy Way to Discuss Multiple Genes Affecting the Same Trait

**DOI:** 10.1371/journal.pbio.1001279

**Published:** 2012-03-06

**Authors:** Michelle Smith

**Affiliations:** School of Biology and Ecology and Research in STEM Education (RiSE) Center, University of Maine, Orono, Maine, United States of America; University of California Berkeley/Joint Genome Institute, United States of America

## Abstract

Developing interactive ways to teach about concepts such as complementation can be difficult. This approach, supported by learning data, uses blind cavefish as an example.

## Summary

Many genetics classroom activities focus on inheritance patterns of a single gene with two different alleles. While valuable, these activities overlook additional areas of genetics research, such as multiple genes controlling a single trait. In this activity, students are introduced to the concept of complementation (see Box 1) and then determine whether blind cave fish from different locations have mutations in the same gene or different genes. Although this activity could be taught in many ways, here it is presented as a lecture with several clicker questions (see Box 2). Student responses are shown to help instructors gauge the range of student answers.

**Box 1. Concepts at a Glance**

**Genetics/molecular biology leads into evolutionary biology**

**Students learn to:**

- Deduce information about genes and alleles from analysis of genetic crosses and patterns of inheritance.
- Interpret complementation tests to determine whether two mutations affect the same gene, and explain the requirements and the basis for these tests.

**Target age group:**

The target age group is an undergraduate genetics course for majors, although this activity could also be part of a high school biology unit on mutations. The genetic alteration of DNA through mutations and the inheritance of mutations is part of the National Academy of Sciences Framework for K-12 Education [7].

**Box 2. Using the Peer Instruction Method in the Classroom**

During lecture, I use the peer instruction method of asking clicker questions [8]. Students vote on an answer choice, talk to their peers, and vote again. Students do not see the distribution of answers until after they talk to their peers and vote again since knowledge of individual voting results can impact peer discussion [9]. Here, I report the votes that students make both before and after discussion with peers so that teachers can follow the impact peer discussion has on student learning [10].

## Introduction

Although there are many examples of genetics activities involving monohybrid and dihybrid crosses, it can be difficult to find activities that focus on more than one gene and a non-simple inheritance pattern. Here, I present an activity that asks students to consider whether multiple genes are responsible for blindness in the Mexican cavefish *Astyanax mexicanus*. These cavefish live in a series of unconnected caves in northeastern Mexico, have been blind for millennia, and can breed with sighted surface fish (reviewed in [1]). In this activity, students answer questions to determine the following: are mutations in the same gene or different genes responsible for blindness in separated cavefish?

I have taught this concept using a variety of active learning strategies. Here, I present the information as an interactive lecture with multiple-choice clicker questions (see Box 3), but this activity could also be taught as a small group activity, assigned as homework, or changed so students would be answering only open-response questions. The advantage of presenting the activity as a lecture with multiple-choice clicker questions is that I can provide quantitative data about how students respond, giving instructors an idea of what fraction of students understood the concept as the class progressed. In this paper, I also include examples of homework and exam questions that ask students to further explore this concept (see Box 4).

**Box 3. Teaching Tools Box**

To help instructors present this concept in class, a PowerPoint file of the interactive lecture slides can be found in Supporting File S1.

Movies about how to use clickers in the classroom and a free instructor's guide to the effective use of clickers can be found at http://www.cwsei.ubc.ca/resources/SEI_video.html.

**Box 4. Evaluation Tools**

**Formative assessment questions on complementation** In addition to learning about complementation in lecture, students answer homework questions. The homework assignment asks students to answer easy (Figure 7) and medium (Figure 8) questions similar to the clicker questions, but also asks them a more difficult question (Figure 9) about a complementation table where only a fraction of the complementation results are included. This problem is challenging for students because they have to decide which cross will give them new information that they cannot already acquire from the complementation table.

**Summative assessment of student learning** Several different measures suggest students learn about complementation. First, students were given a complementation question on the exam similar to the difficult homework question (Figure 10) and 88% of the students answered correctly. Second, I gave the Genetics Concept Assessment (GCA) [11] the first day of the class and put the identical questions on the final exam. One question on this assessment asks about complementation (Figure 11). On the pretest, only 44% of the students answer this question correctly, but on the posttest 93% of the students answer this question correctly.

The data shown here are from a large lecture genetics course at a state university (*n* = 120 students). The classes met twice a week for a 90-minute lecture and also included a 50-minute recitation section. During recitation section, students worked in small groups to solve genetics problems. The course included weekly homework, clicker questions in each lecture, and three exams. Student demographic information is included in Table 1.

**Table 1:** Student demographic information.

Category	Demographic Information
Class Standing	18% junior, 74% senior
Ethnicity	63% non-Caucasian
Sex	61% female
Major	62% biology, 23% biochemistry

## Engagement: Introducing Complementation Using Human Deafness

**Figure 1:**
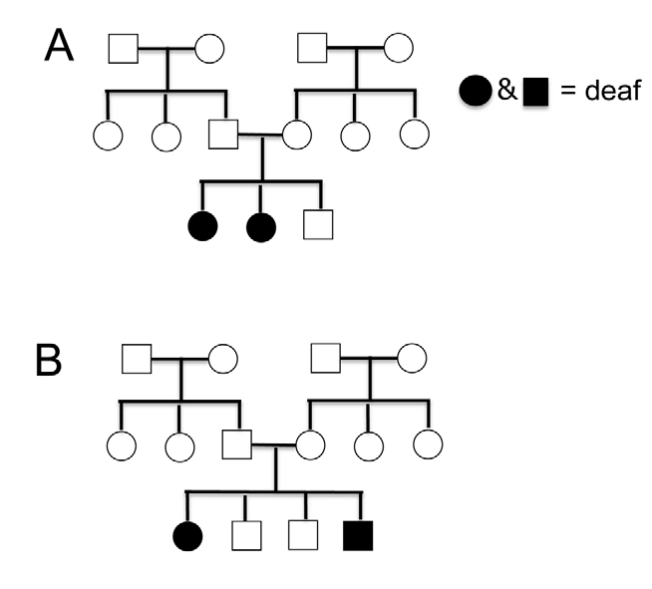
Human pedigrees used to introduce the concept of complementation. One family where several members are deaf is shown in (A), a second family where several members are deaf is shown in (B).

I begin this unit by building on two concepts we have already covered in the course: 1) identifying autosomal inheritance patterns and 2) analyzing pedigrees. Specifically, students analyze a human pedigree on the inheritance of deafness (shaded individuals) to review these concepts (Figure 1A). Human deafness is a good example for introducing students to multi-gene traits because mutations in at least 57 genes cause deafness that is not associated with any other symptoms (http://deafnessvariationdatabase.org). I ask the class to tell me about the inheritance pattern for the pedigree in Figure 1A. Students are quick to volunteer that this pedigree is more consistent with an autosomal recessive inheritance pattern. I then ask students to tell me about the inheritance pattern in the Figure 1B pedigree. Students again say that the pedigree is more consistent with an autosomal recessive inheritance pattern.

Next, I tell students that a deaf person from the family in Figure 1A has a child with a deaf person from the family in Figure 1B, and that child can hear. This result is surprising because if we were considering a simple Mendelian inheritance pattern, two people who are deaf because of autosomal recessive mutations would have a 100% chance of having a deaf child. I then ask the class: “how can you explain the child from the mating between the two pedigrees?” Students often suggest that the sperm or egg that created the child had a spontaneous mutation that made a mutant allele normal (a possibility, but not very likely) or that the child has the genetic mutations but for some environmental reason, s/he is not deaf (another possibility, so I respond by saying let us assume all individuals with the mutation are deaf). Sometimes a student will suggest that the parents have mutations in two different genes.

**Figure 2:**
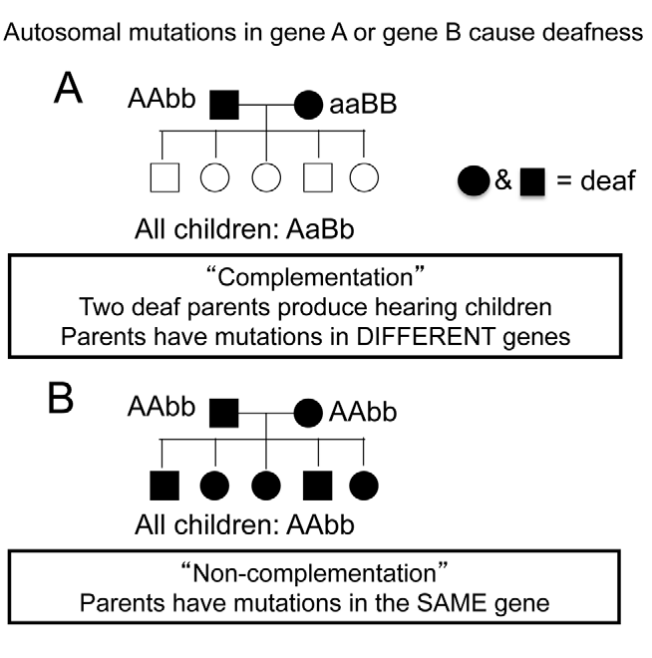
A slide introducing students to the terms complementation and non-complementation in the context of the human deafness example.

I now introduce students to the terms “complementation” and “non-complementation” using the slides in Figure 2. I state that an example of complementation occurs when two deaf parents produce hearing children. These results suggest that the parents have mutations in DIFFERENT genes. I represent the two genes using the letters A and B (Figure 2A). Then I contrast complementation with non-complementation (Figure 2B). Here, two deaf parents produce all deaf children. This result suggests that parents have recessive mutations in the SAME gene, represented by the letter B.

## Inquiry: Engaging Students in Applying Complementation to Blind Cavefish

To apply the concept of complementation, students study eye development in the Mexican blind cavefish. These blind cavefish populations, which evolved independently from sighted surface fish at different times, are found in caves throughout northeastern Mexico (reviewed in [1],[2]). The explanation for the evolution of blindness remains in dispute [3]. When students want to know more about hypotheses for why cavefish lost their sight, I encourage them to read more in several review articles [1]–[3] and an article written for a general audience [4].

To engage students in the process of inquiry, I show a screen shot of Borowsky's article “Restoring Sight in Blind Cavefish” [5] so students can see that the concepts they are learning about in class are studied by geneticists. I also show a screen shot of a National Geographic news story that begins “It's a miracle! Blind cavefish, despite having adapted to their lightless environment for more than a million years, can produce sighted offspring in just a single generation…” [6]. I tell students that no miracle is required for these results, just genetics.

## Investigation: Applying Knowledge of Complementation to Blind Fish

**Figure 3:**
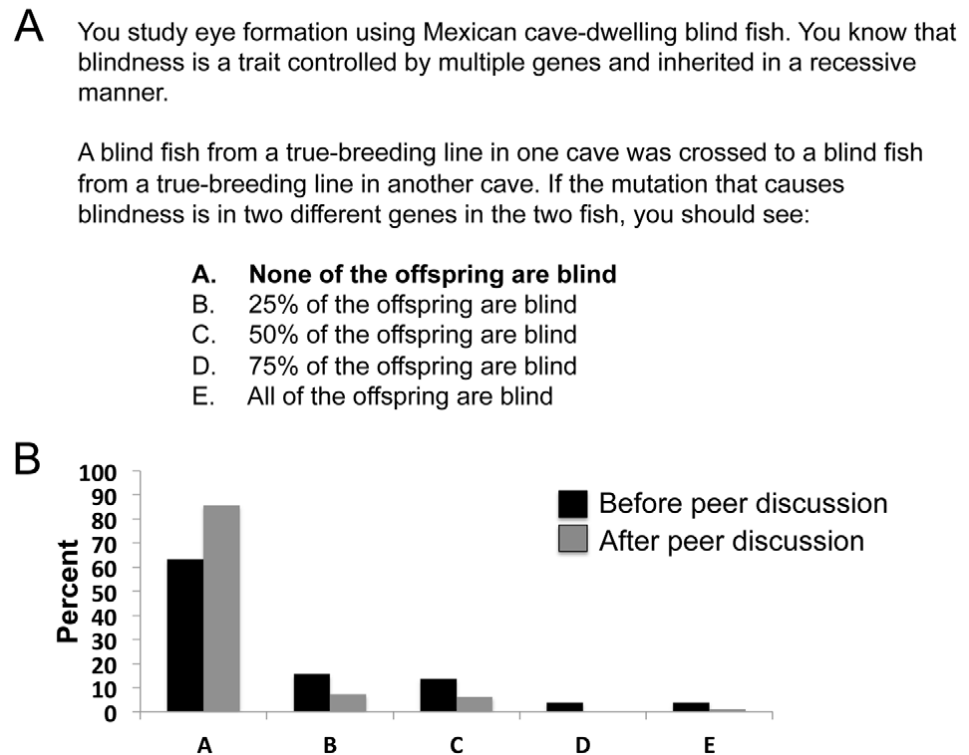
Clicker question to determine whether students can apply what they learned about complementation to an example of blind cavefish. (A) The text of the question with the correct answer in bold. (B) Student responses both before and after peer discussion (*n* = 103 students).

Next, I ask a clicker question that tests whether students can apply what they learned about complementation in human deafness to the cavefish example (Figure 3A). This question asks: if you mate two blind fish that have recessive mutations in two different genes, what percentage of their offspring will be blind? When students answer the question before talking with their peers, 63% of the students correctly answer that none of the offspring will be blind (Figure 3B). Interestingly, the most common incorrect answers are b) 25% or c) 50%, suggesting that students are answering the question as if they were solving a simple Mendelian inheritance monohybrid cross problem. After the students talk to their peers, 86% of the students answer correctly (Figure 3B), indicating that peer discussion alone is improving student performance.

## Clarification: Constructing a Complementation Table

**Figure 4:**
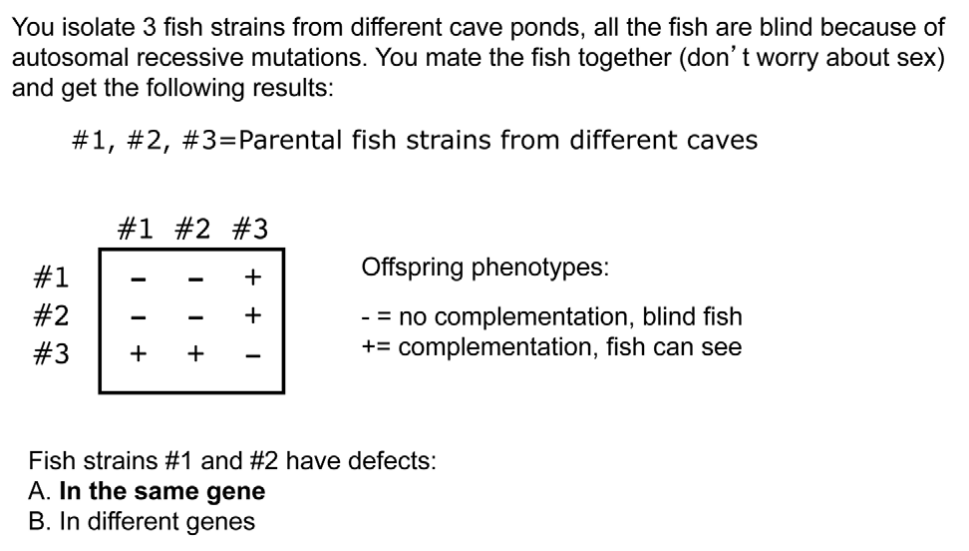
Clicker question to introduce the notation used in a complementation table.

Next, I introduce students to the complementation table. Complementation tables track the outcomes of mating fish from different strains, and they are useful in determining whether the mutations are in the same gene or different genes (Figure 4). On the outside of the table you put a unique identifier for each fish strain collected from the different caves (for example strain #1, strain #2, etc.) and inside the table you write either a + or a −. A + means that complementation occurred (the genes are different), and a − indicates that complementation did not occur (the genes are the same). To confirm that students are following, I ask them the clicker question in Figure 4. Even before peer discussion, 94% of the class answered that mating two strains with the result that all the offspring are blind indicates that the mutations must be in the same gene.

## Interpreting Results: How Many Genes Influence a Trait?

Geneticists can use complementation tables to determine the minimum number of genes influencing a trait. This number can be obtained by combining the mutations that do not complement and making sure all strains are accounted for. In the example in Figure 4, there are a minimum of two genes controlling eye development in these fish. Strains #1 and #2 have a mutation in one gene and strain #3 has a mutation in a second gene.

**Figure 5:**
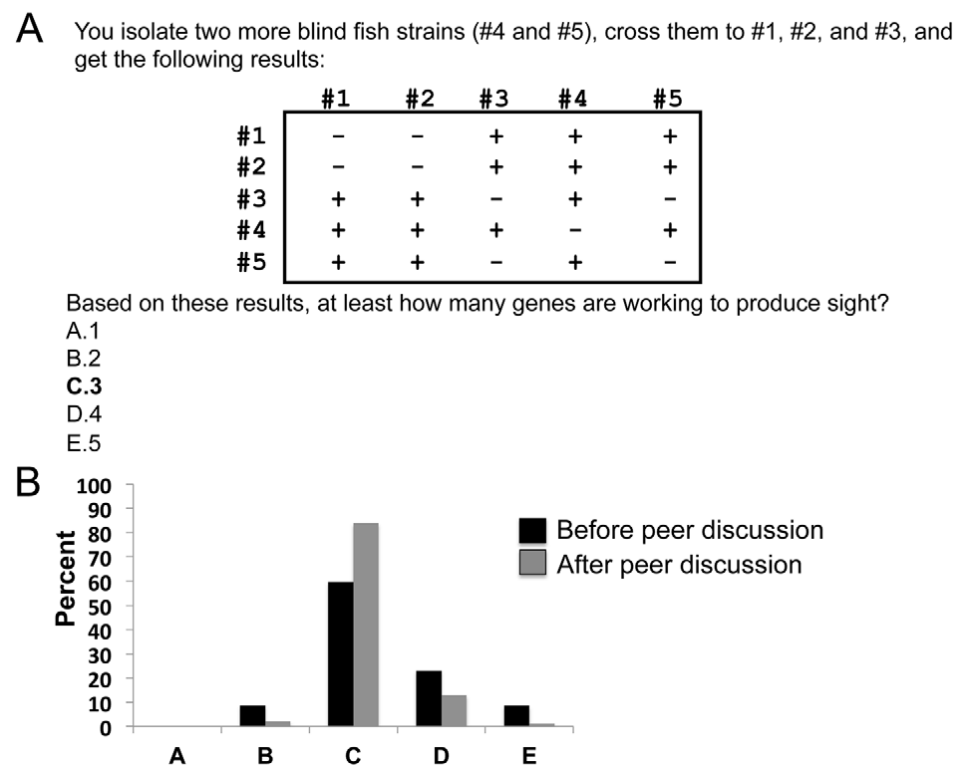
Clicker question to determine whether students can interpret the results of several complementation tests. (A) The text of the question with the correct answer in bold. (B) Student responses both before and after peer discussion (*n* = 103 students).

Next, I expand the table in Figure 4 to five different strains and have students determine how many genes produce sight (Figure 5A). Here, strains #1 and #2 have a mutation in one gene, strains #3 and #5 have a mutation in another gene, and strain #4 complements the other strains. The results suggest that at least three genes are working to produce sight. Before peer discussion only 60% of the students answer this question correctly, but this number increases to 84% after peer discussion (Figure 5B).

## Exploring: What Are the Limits of Complementation Testing?

**Figure 6:**
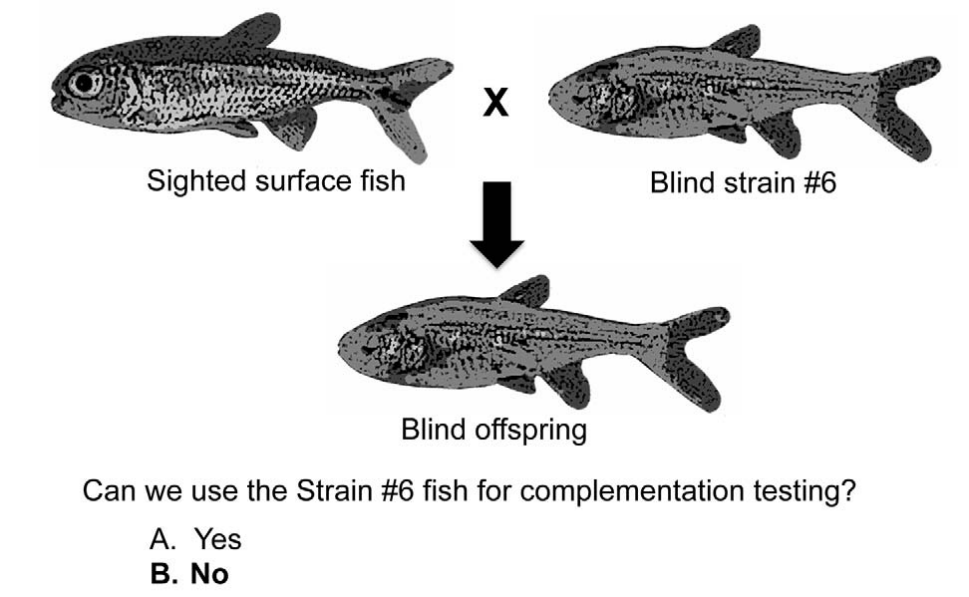
Clicker question about the limits of complementation testing.

Now I use another clicker question to have students consider a new blind fish strain, strain #6. Strain #6 is mated to a sighted surface fish and all the offspring fish are blind (Figure 6). The question asks: can the strain #6 fish be used for complementation testing? The answer is no, because the strain #6 fish has a dominant version of a gene involved in eyesight. Anytime a fish from strain #6 is mated to any blind fish, the offspring will be blind. Therefore, geneticists cannot accurately score complementation versus non-complementation using a fish that has a dominant mutation. Before talking with peers, only 54% of the students say the strain #6 fish cannot be used for complementation testing, but after peer discussion, this number increases to 77%. As with the previous questions, discussion with peers improves student performance.

**Figure 7:**
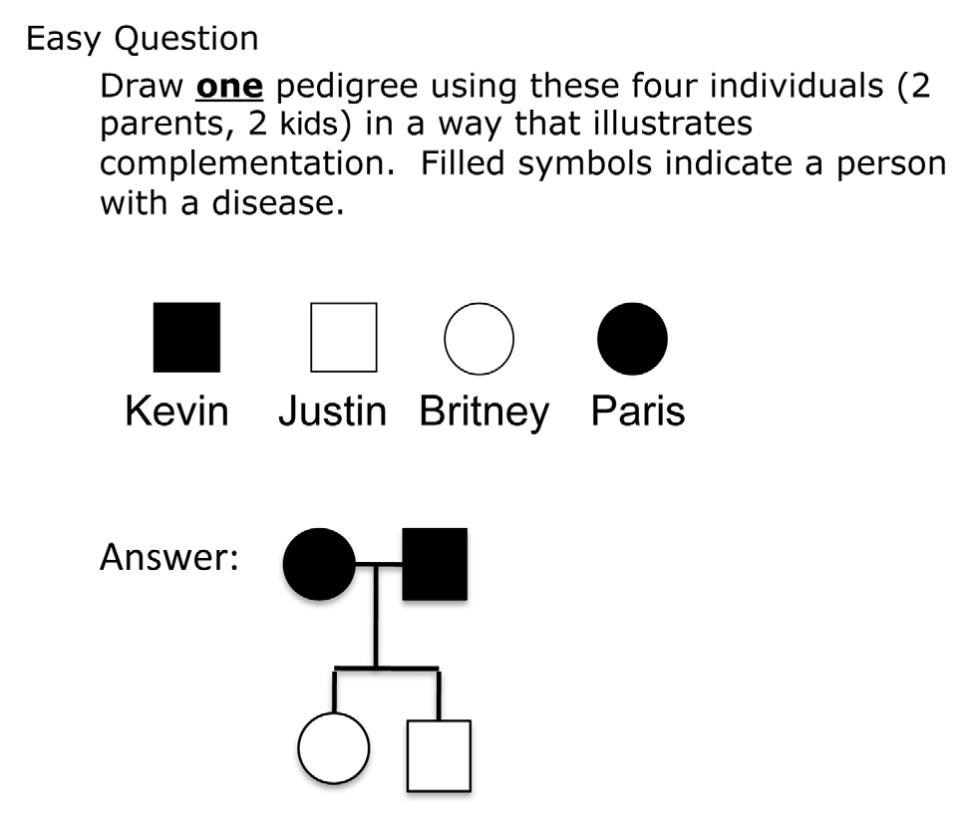
Easy complementation homework question.

**Figure 8:**
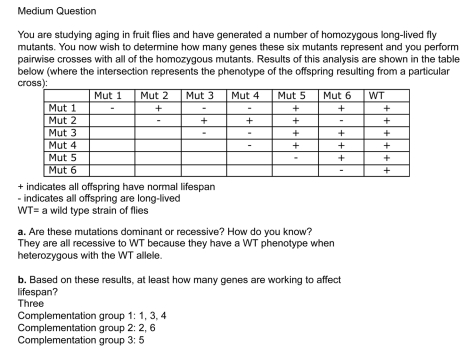
Medium complementation homework question.

**Figure 9:**
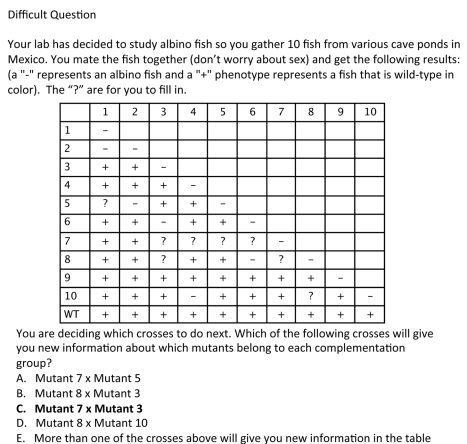
Difficult complementation homework question. The correct answer is marked in bold font.

**Figure 10:**
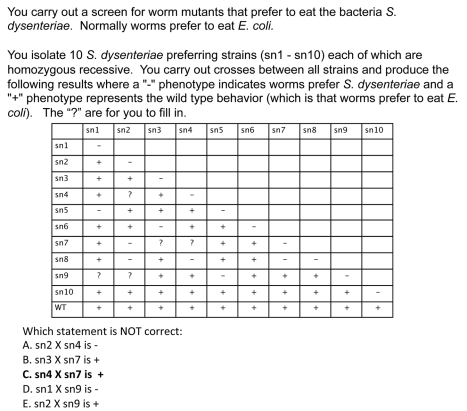
Exam question on complementation. The correct answer is marked in bold font.

**Figure 11:**
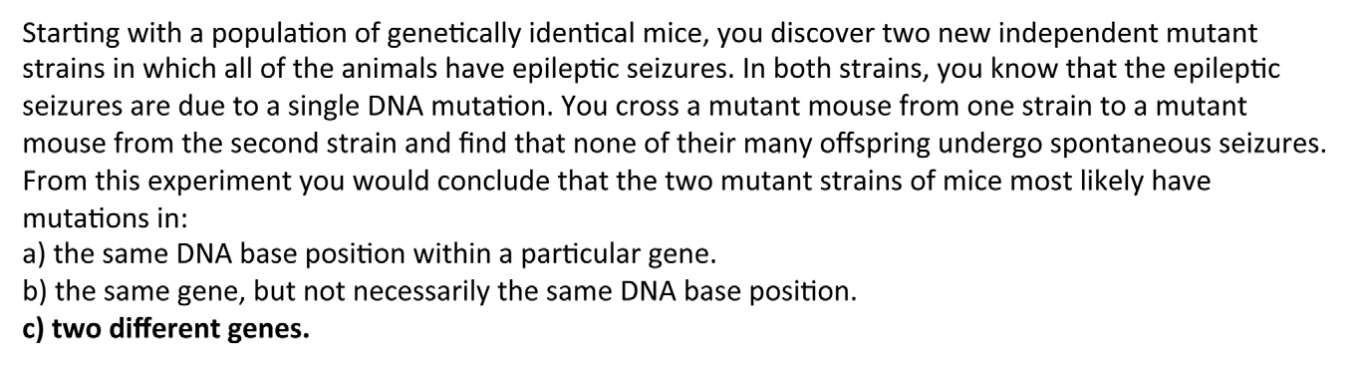
GCA question on complementation. The correct answer is marked in bold font.

## Conclusion

The activity described here helps students learn how one trait, such as deafness or blindness, can be affected by mutations in more than one gene and how geneticists can determine the minimum number of genes involved. Students also learn why strains with dominant mutations cannot be used for complementation testing. The clicker question results consistently indicate that students learn about complementation by talking over questions with their peers and there is evidence that this learning serves them well on exams and a genetics concept assessment (see Box 4).

### IRB Statement

Approval to evaluate student clicker, exam, and pretest and posttest responses (exempt status, Protocol No. 39014) was granted by the Institutional Review Board, University of Washington.

## Supporting Information

Supporting File S1 (PPT)
