# Correction: A Fishy Way to Discuss Multiple Genes Affecting the Same Trait

**DOI:** 10.1371/annotation/301bb6ce-46b7-42f1-8936-8ab33772b732

**Published:** 2012-06-04

**Authors:** Michelle Smith

The Funding information was incorrect. The correct Funding is: This material is based upon work supported in part by the National Science Foundation under Grant #0962805. The funders had no role in study design, data collection and analysis, decision to publish, or preparation of the manuscript. 

