# Supplementary material for: A Fishy Way to Discuss Multiple Genes Affecting the Same Trait
Source: PLoS Biol. 2012 Mar 6;10(3):e1001279. doi: 10.1371/journal.pbio.1001279 (PMC3295817; doi:10.1371/journal.pbio.1001279)
Supplement: Supporting File S1 — (PPT) [file pbio.1001279.s001.ppt]

## Slide 1
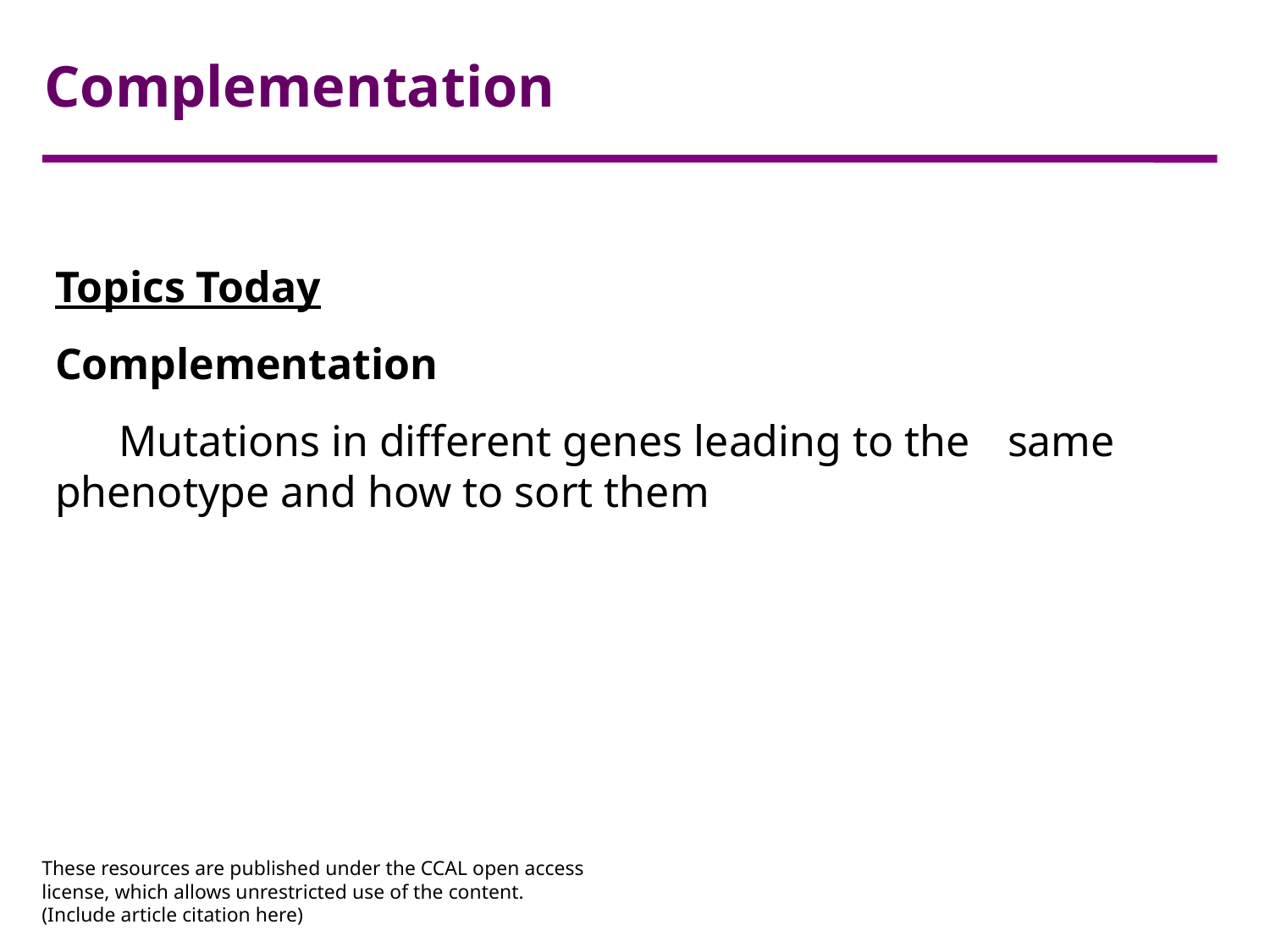

Complementation
Topics Today
Complementation
	Mutations in different genes leading to the 	same phenotype and how to sort them
These resources are published under the CCAL open access license, which allows unrestricted use of the content. (Include article citation here)

## Slide 2
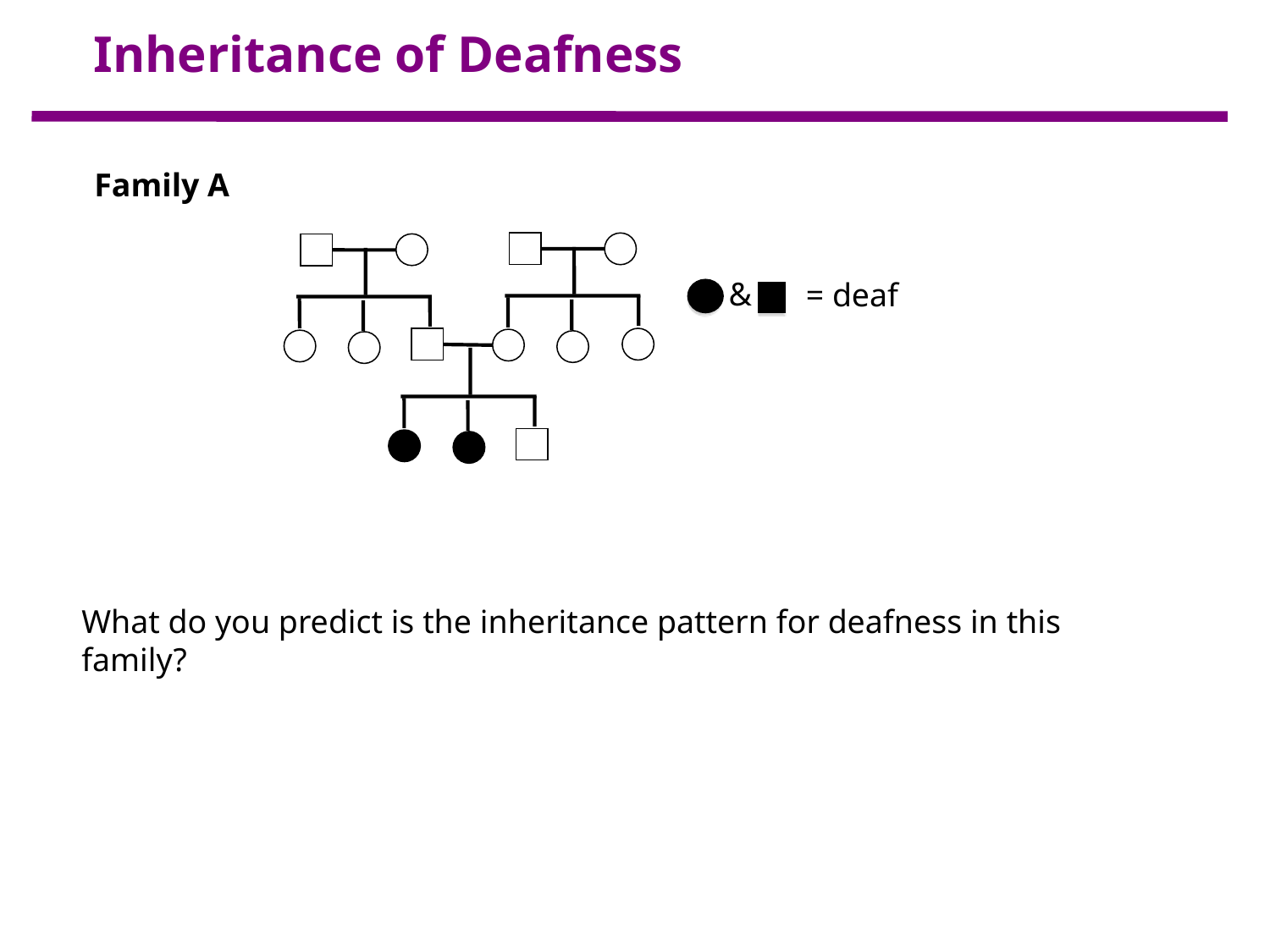

Inheritance of Deafness
Family A
&
= deaf
What do you predict is the inheritance pattern for deafness in this family?

## Slide 3
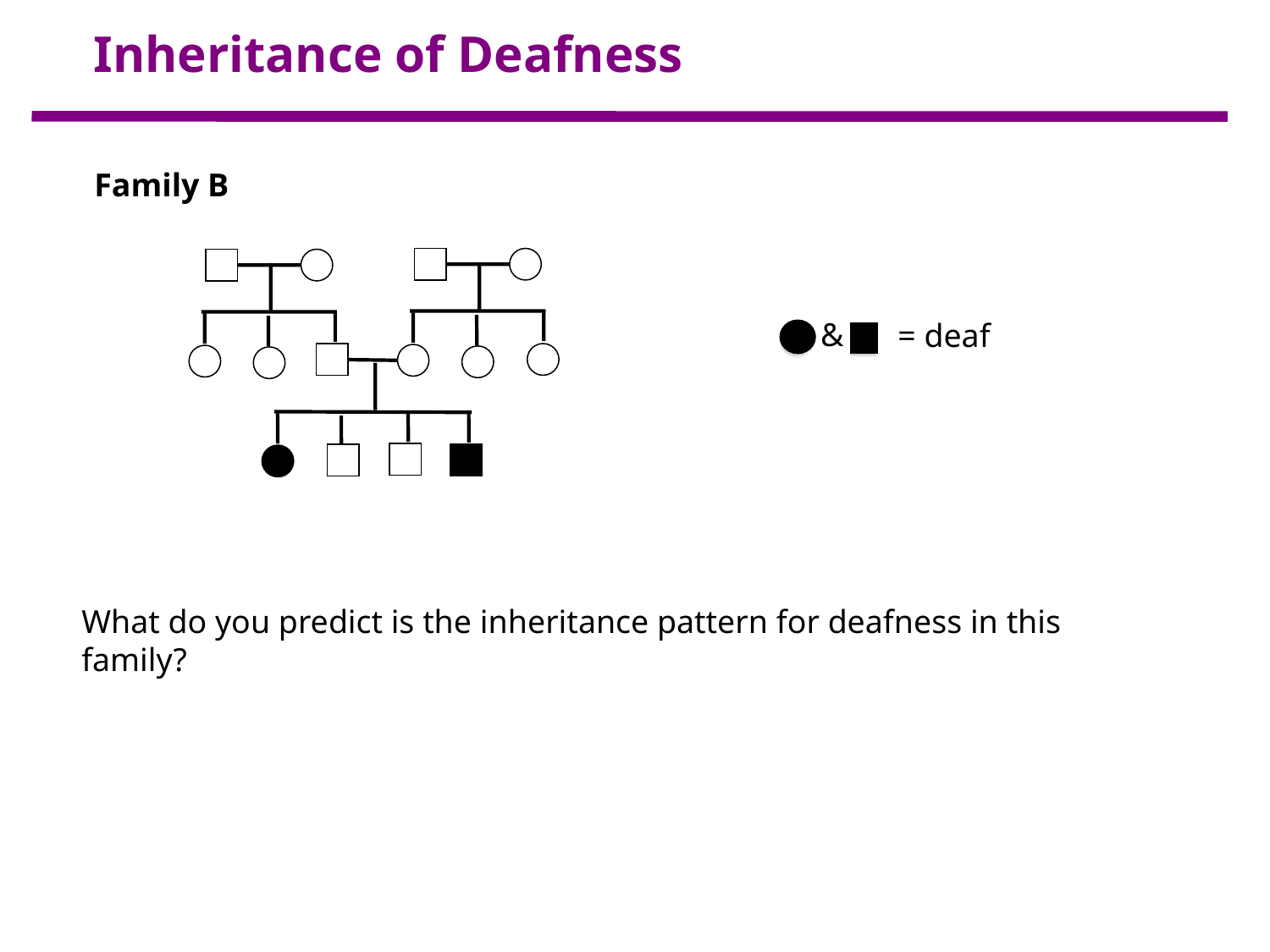

Inheritance of Deafness
Family B
&
= deaf
What do you predict is the inheritance pattern for deafness in this family?

## Slide 4
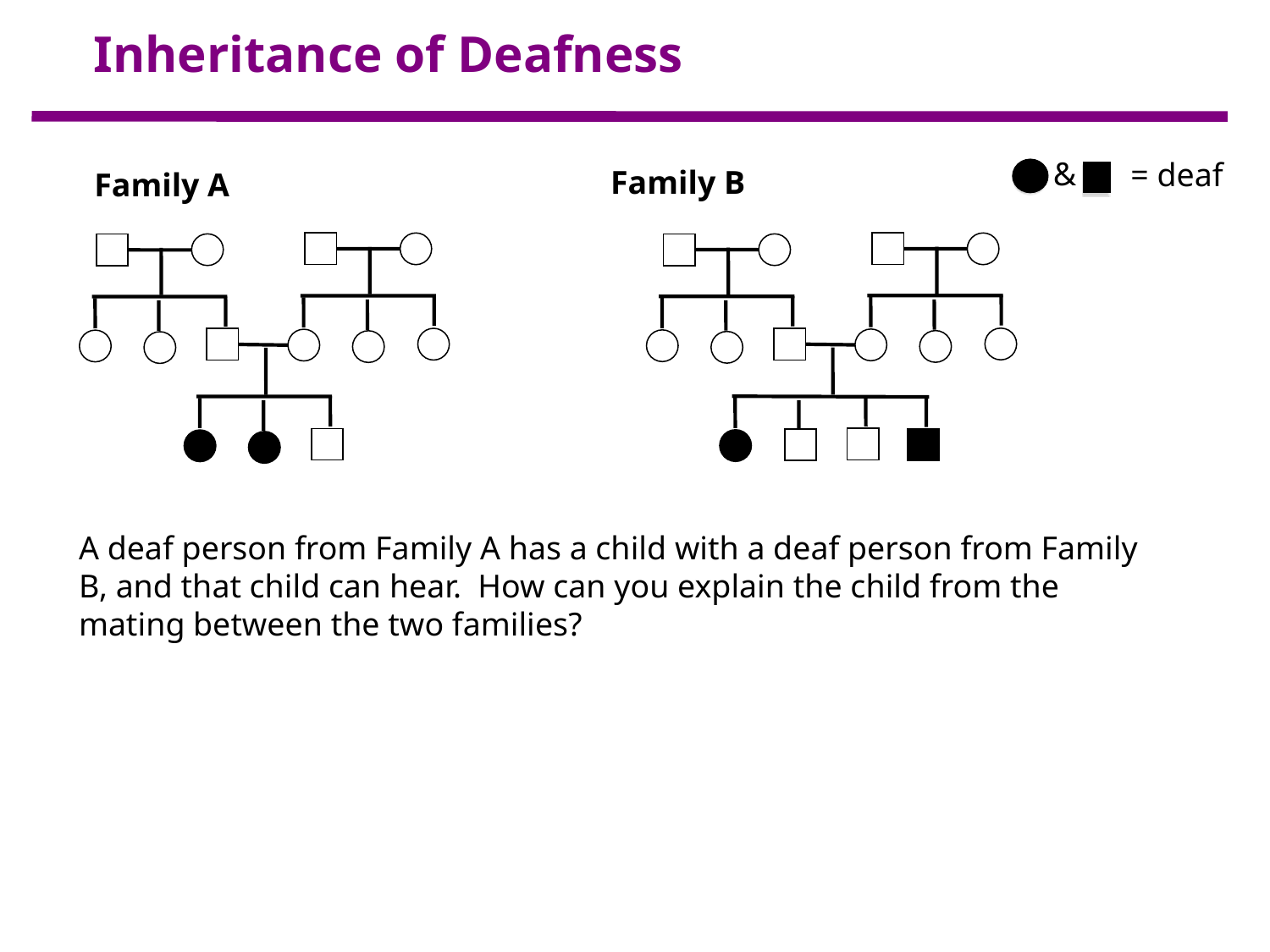

Inheritance of Deafness
&
= deaf
Family B
Family A
A deaf person from Family A has a child with a deaf person from Family B, and that child can hear. How can you explain the child from the mating between the two families?

## Slide 5
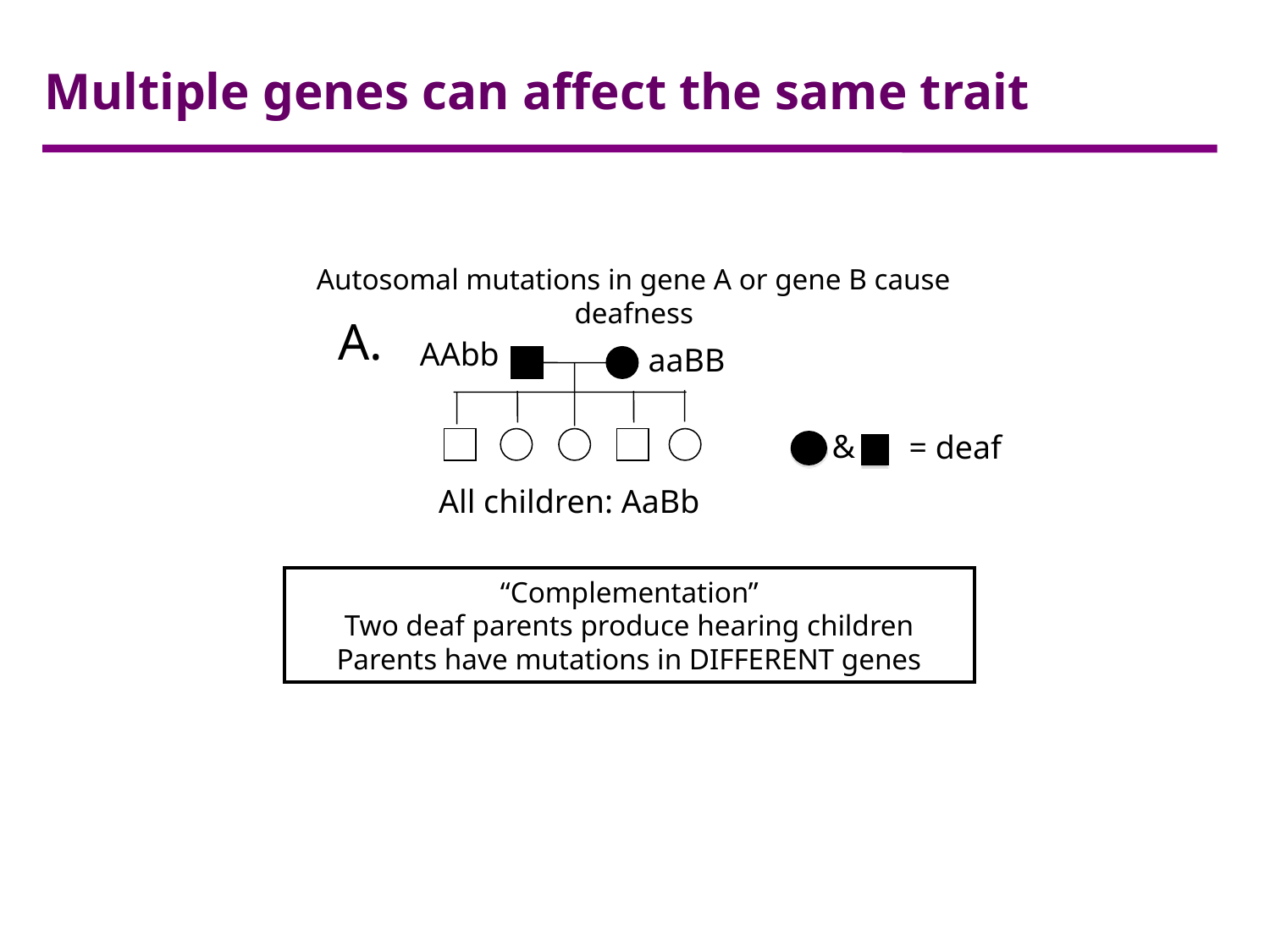

# Multiple genes can affect the same trait
Autosomal mutations in gene A or gene B cause deafness
A.
AAbb
aaBB
&
= deaf
All children: AaBb
“Complementation”
Two deaf parents produce hearing children
Parents have mutations in DIFFERENT genes

## Slide 6
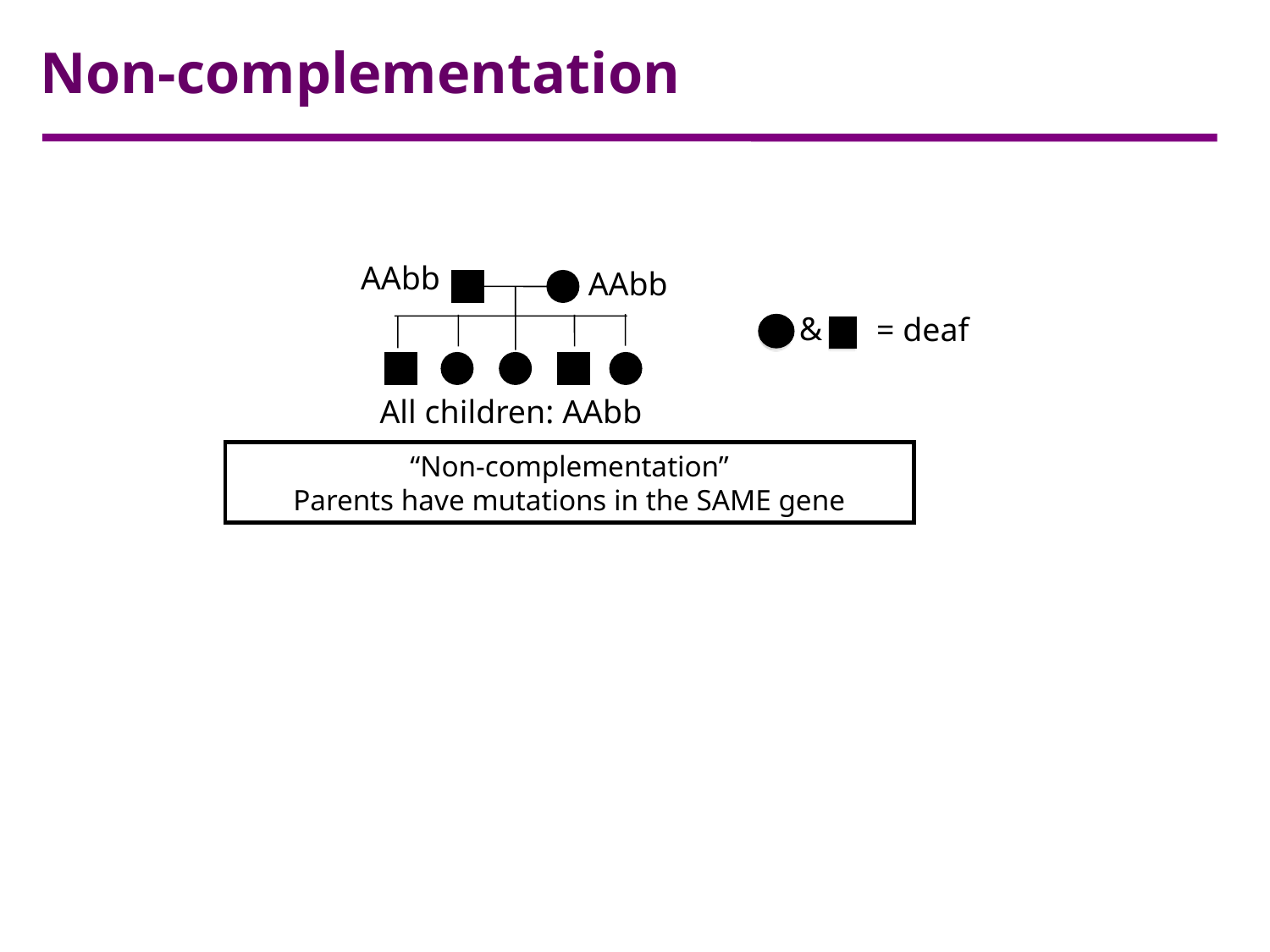

Non-complementation
AAbb
AAbb
&
= deaf
All children: AAbb
“Non-complementation”
Parents have mutations in the SAME gene

## Slide 7
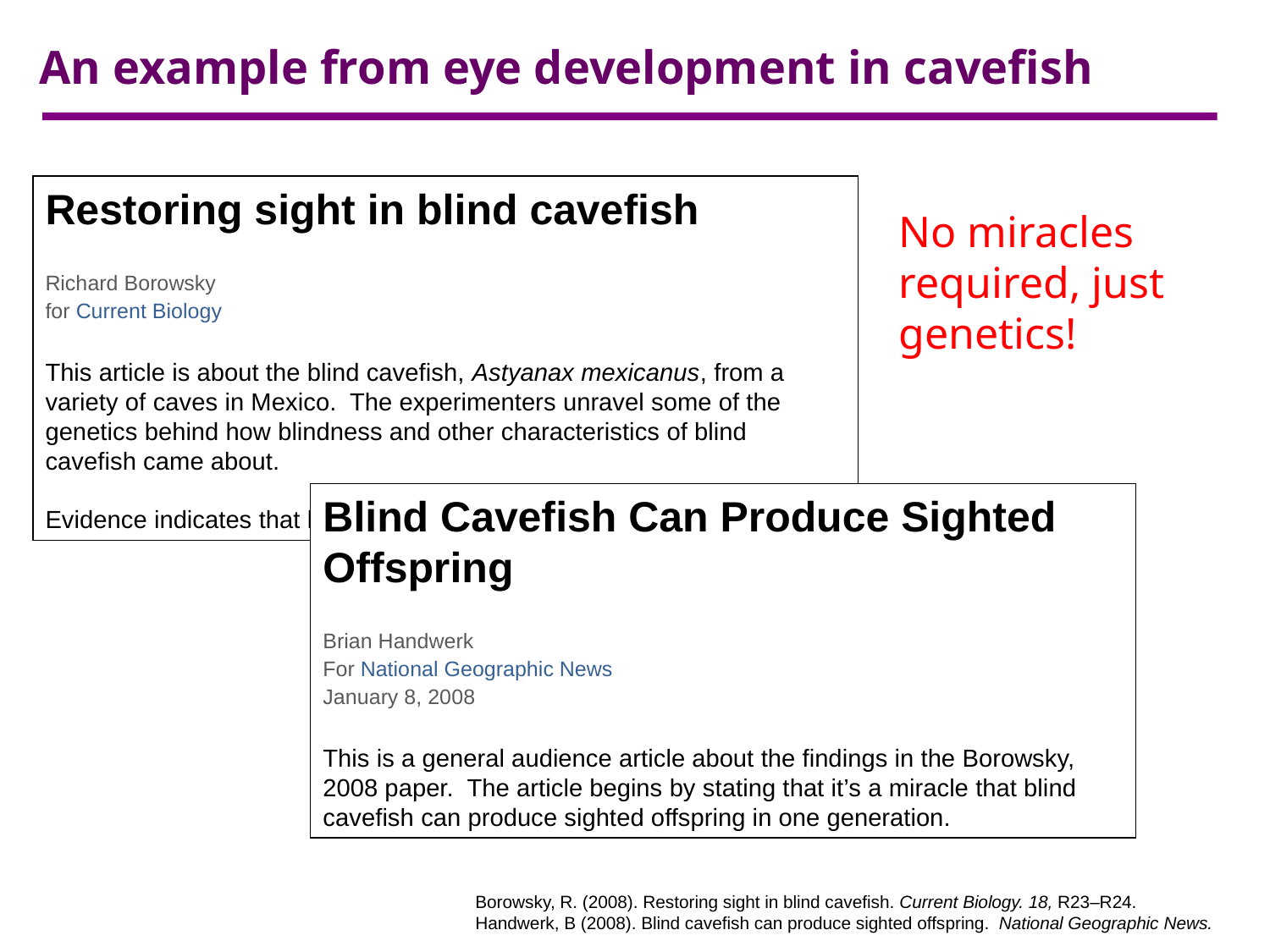

An example from eye development in cavefish
Restoring sight in blind cavefish
Richard Borowsky
for Current Biology
This article is about the blind cavefish, Astyanax mexicanus, from a variety of caves in Mexico. The experimenters unravel some of the genetics behind how blindness and other characteristics of blind cavefish came about.
Evidence indicates that blindness arose more than once among
No miracles required, just genetics!
Blind Cavefish Can Produce Sighted Offspring
Brian Handwerk
For National Geographic News
January 8, 2008
This is a general audience article about the findings in the Borowsky, 2008 paper. The article begins by stating that it’s a miracle that blind cavefish can produce sighted offspring in one generation.
Borowsky, R. (2008). Restoring sight in blind cavefish. Current Biology. 18, R23–R24.
Handwerk, B (2008). Blind cavefish can produce sighted offspring. National Geographic News.

## Slide 8
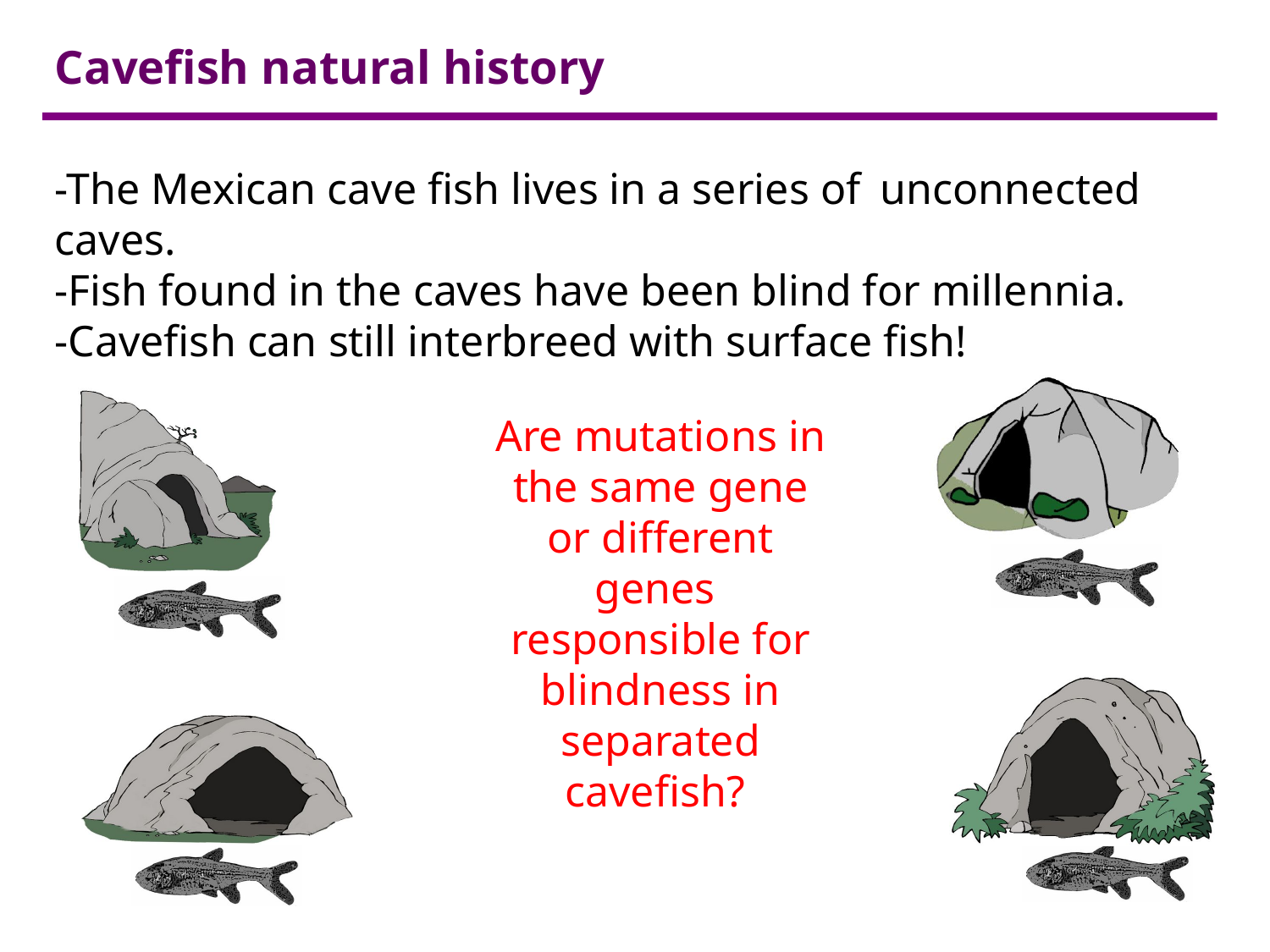

Cavefish natural history
-The Mexican cave fish lives in a series of 	unconnected caves.
-Fish found in the caves have been blind for millennia.
-Cavefish can still interbreed with surface fish!
Are mutations in the same gene or different genes responsible for blindness in separated cavefish?

## Slide 9
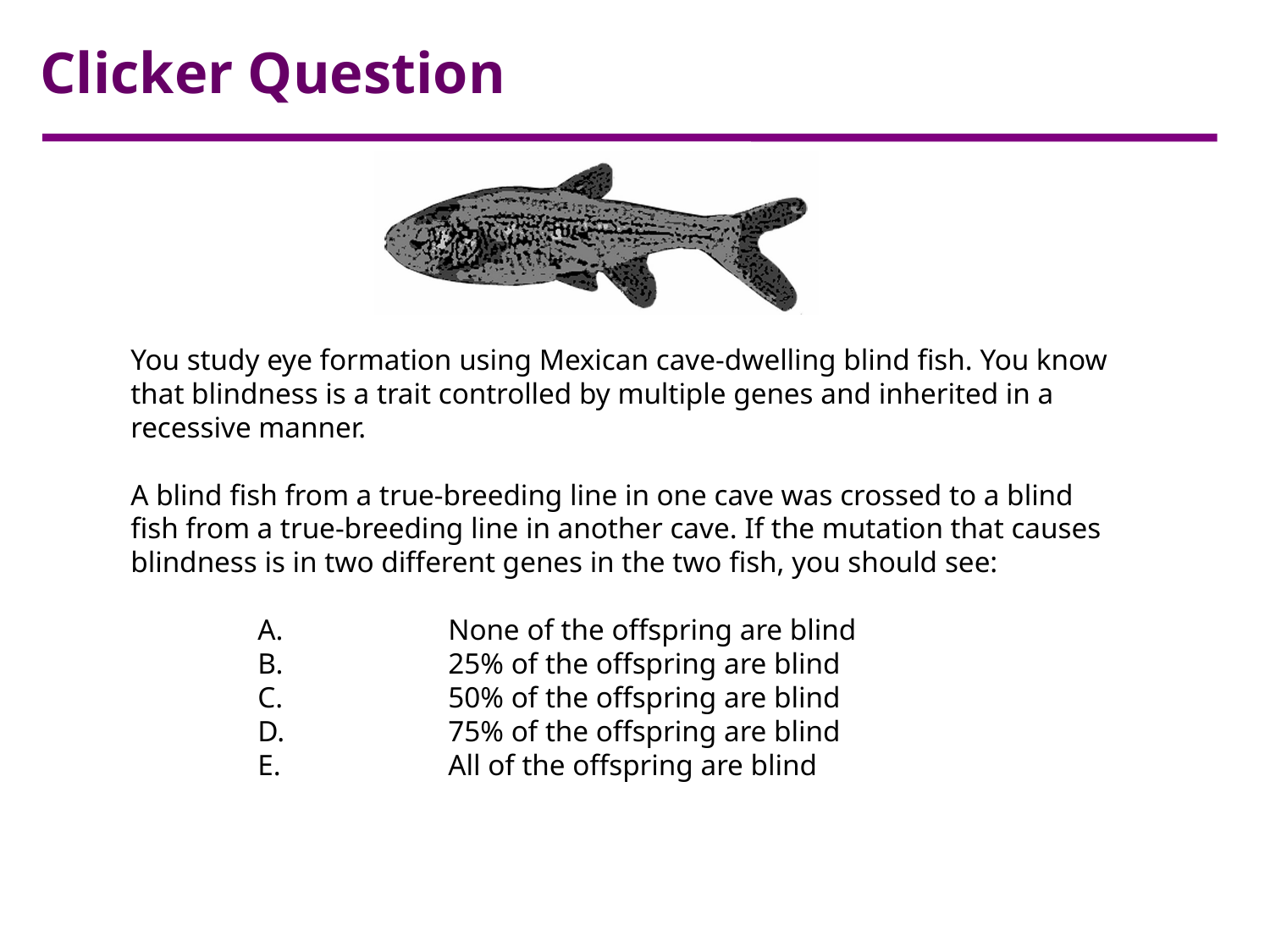

Clicker Question
You study eye formation using Mexican cave-dwelling blind fish. You know that blindness is a trait controlled by multiple genes and inherited in a recessive manner.
A blind fish from a true-breeding line in one cave was crossed to a blind fish from a true-breeding line in another cave. If the mutation that causes blindness is in two different genes in the two fish, you should see:
A.	None of the offspring are blind
B.	25% of the offspring are blind
C.	50% of the offspring are blind
D.	75% of the offspring are blind
E.	All of the offspring are blind

## Slide 10
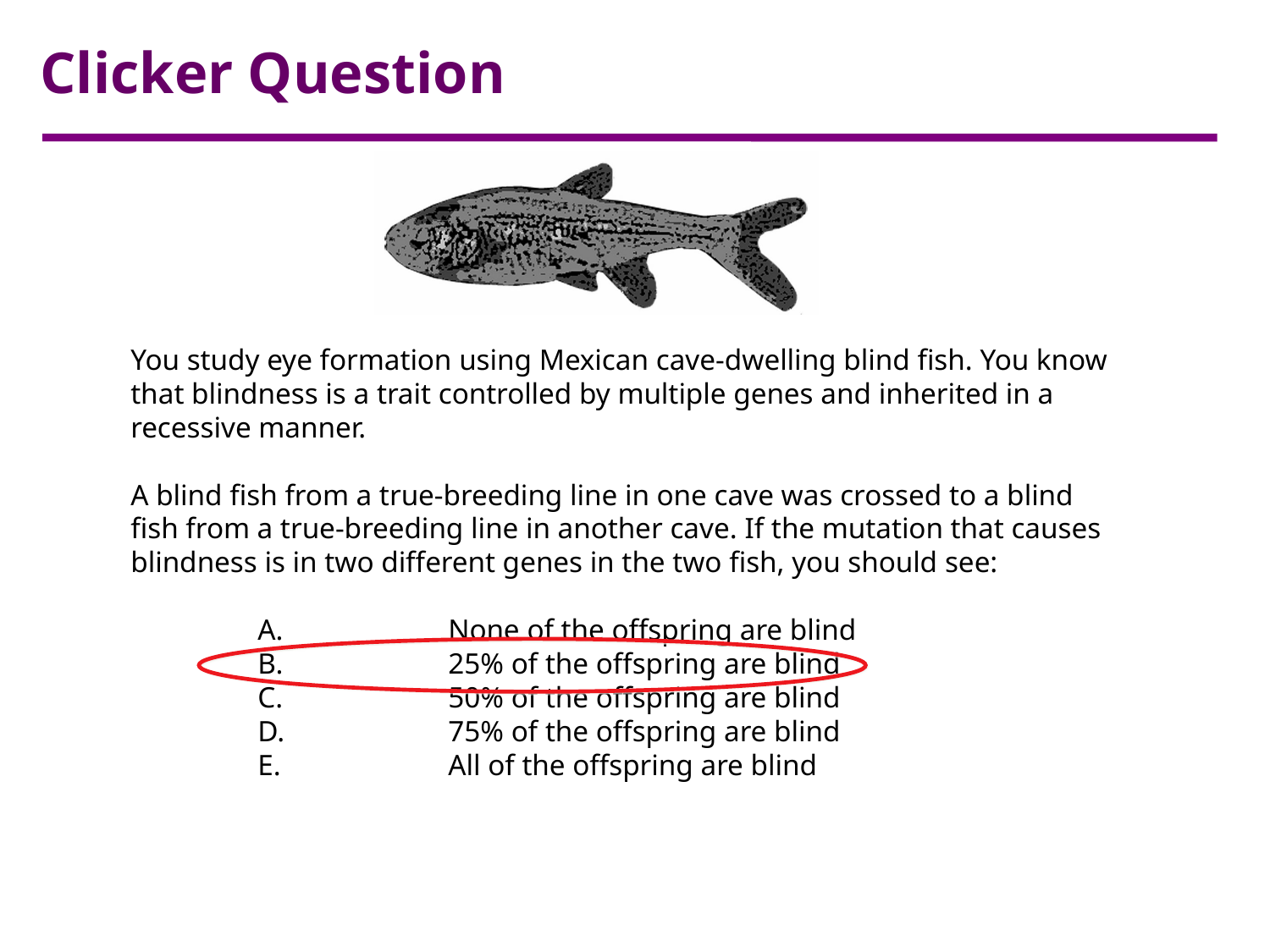

Clicker Question
You study eye formation using Mexican cave-dwelling blind fish. You know that blindness is a trait controlled by multiple genes and inherited in a recessive manner.
A blind fish from a true-breeding line in one cave was crossed to a blind fish from a true-breeding line in another cave. If the mutation that causes blindness is in two different genes in the two fish, you should see:
A.	None of the offspring are blind
B.	25% of the offspring are blind
C.	50% of the offspring are blind
D.	75% of the offspring are blind
E.	All of the offspring are blind

## Slide 11
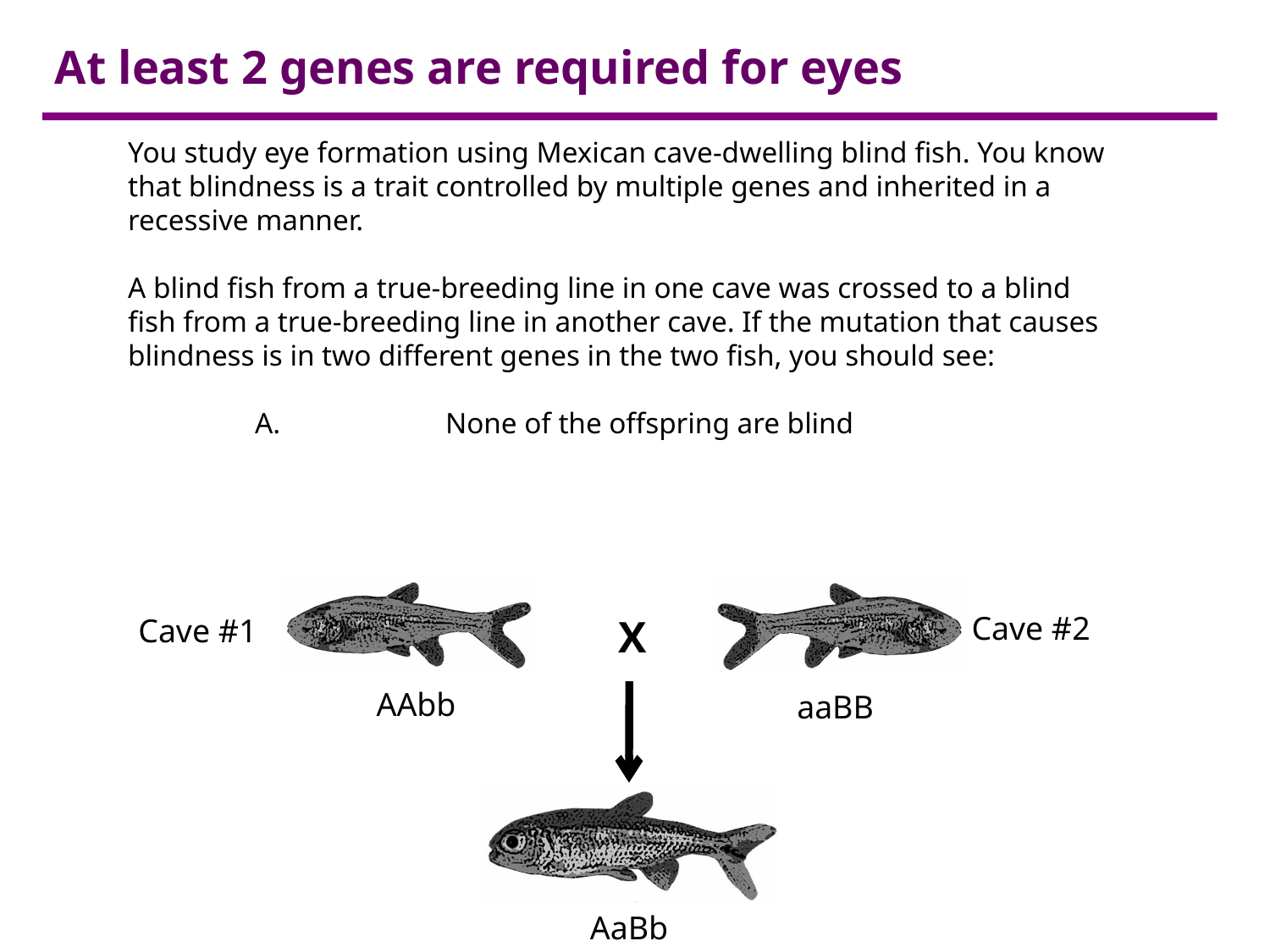

At least 2 genes are required for eyes
You study eye formation using Mexican cave-dwelling blind fish. You know that blindness is a trait controlled by multiple genes and inherited in a recessive manner.
A blind fish from a true-breeding line in one cave was crossed to a blind fish from a true-breeding line in another cave. If the mutation that causes blindness is in two different genes in the two fish, you should see:
A.	None of the offspring are blind
Cave #2
Cave #1
X
AAbb
aaBB
AaBb

## Slide 12
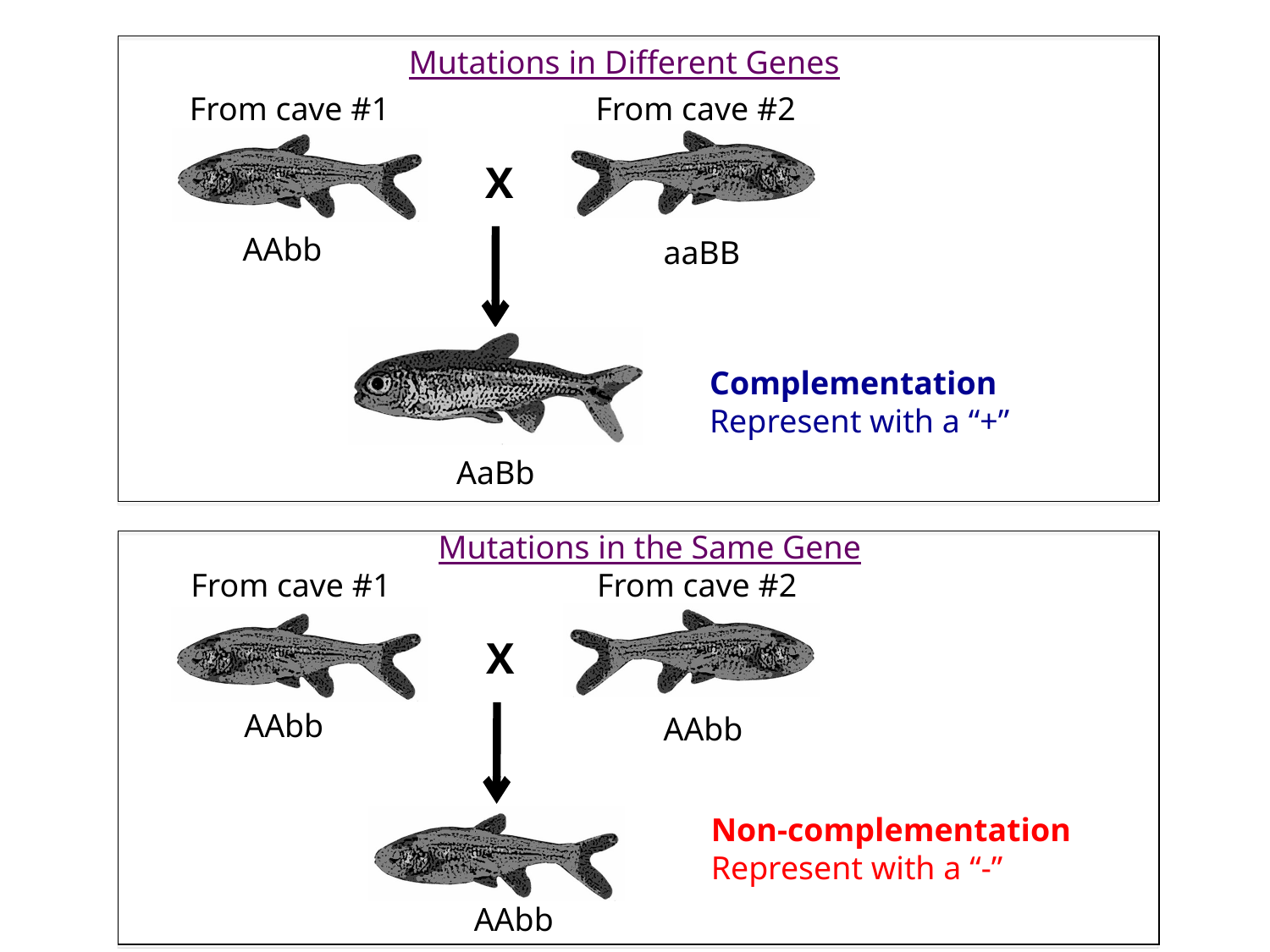

Mutations in Different Genes
From cave #1
From cave #2
X
AAbb
aaBB
Complementation
Represent with a “+”
AaBb
Mutations in the Same Gene
From cave #1
From cave #2
X
AAbb
AAbb
Non-complementation
Represent with a “-”
AAbb

## Slide 13
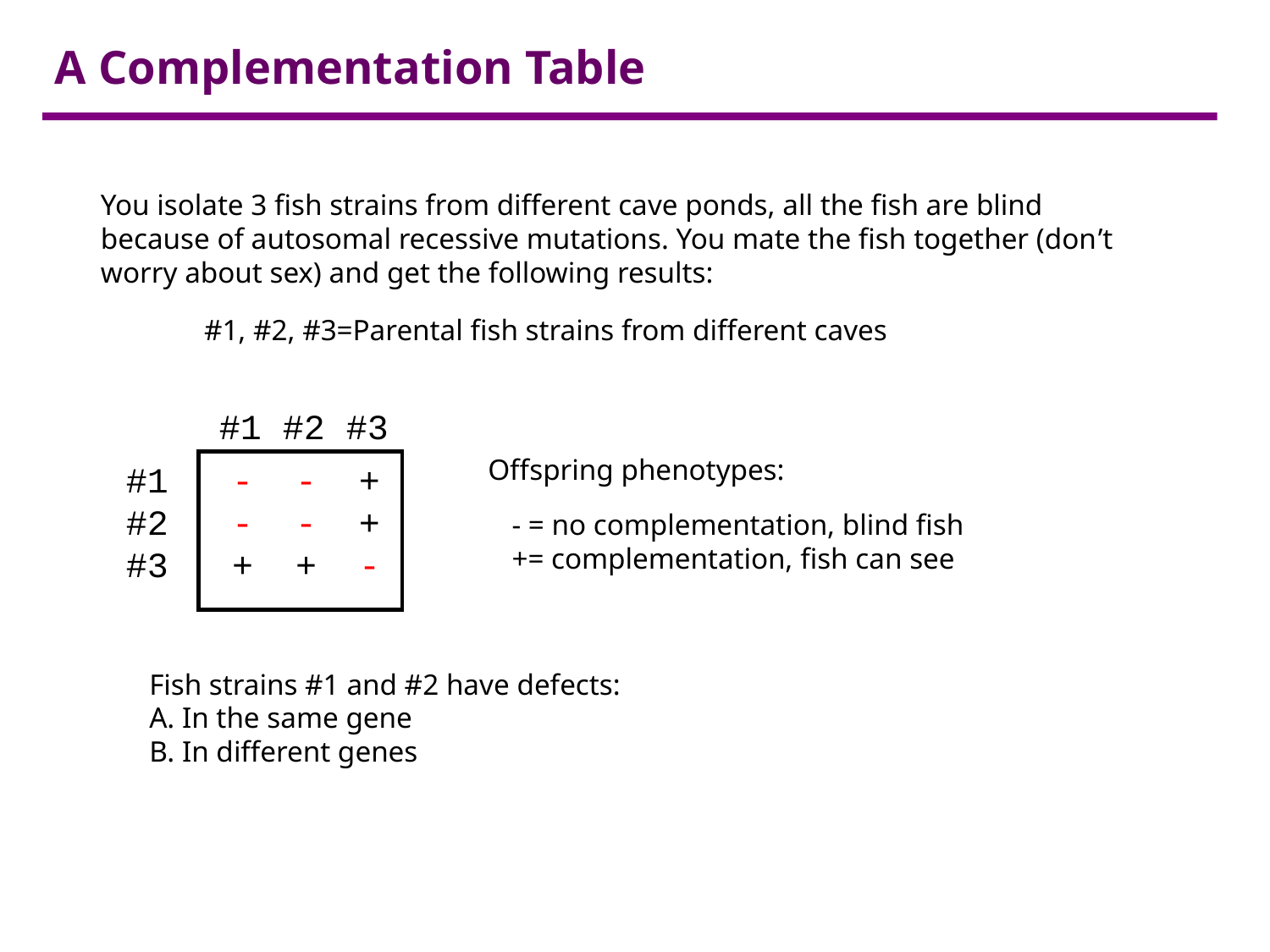

A Complementation Table
You isolate 3 fish strains from different cave ponds, all the fish are blind because of autosomal recessive mutations. You mate the fish together (don’t worry about sex) and get the following results:
#1, #2, #3=Parental fish strains from different caves
#1	#2 #3
Offspring phenotypes:
- = no complementation, blind fish
+= complementation, fish can see
#1 - - +
#2 - - +
#3 + + -
Fish strains #1 and #2 have defects:
A. In the same gene
B. In different genes

## Slide 14
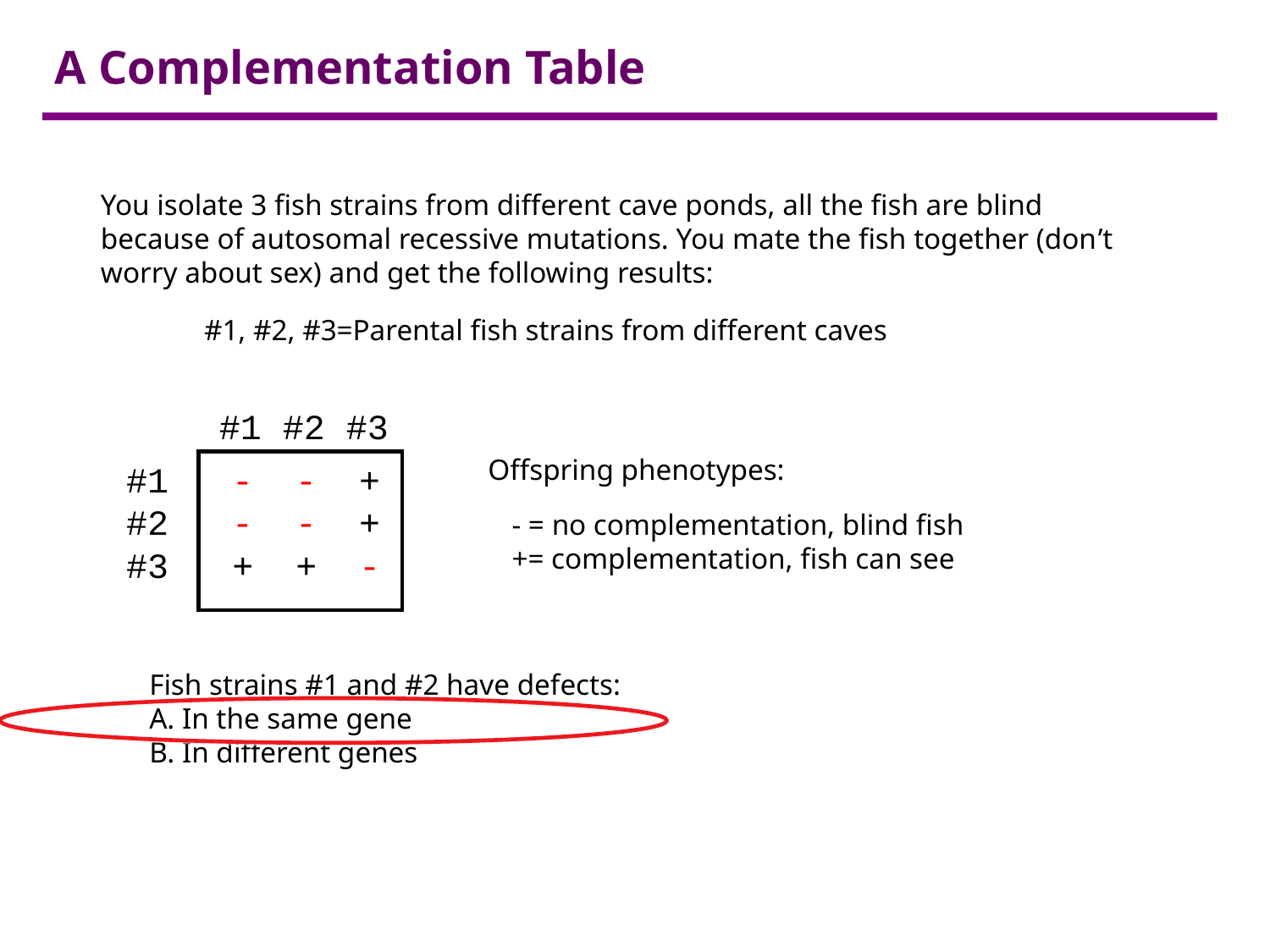

A Complementation Table
You isolate 3 fish strains from different cave ponds, all the fish are blind because of autosomal recessive mutations. You mate the fish together (don’t worry about sex) and get the following results:
#1, #2, #3=Parental fish strains from different caves
#1	#2 #3
Offspring phenotypes:
- = no complementation, blind fish
+= complementation, fish can see
#1 - - +
#2 - - +
#3 + + -
Fish strains #1 and #2 have defects:
A. In the same gene
B. In different genes

## Slide 15
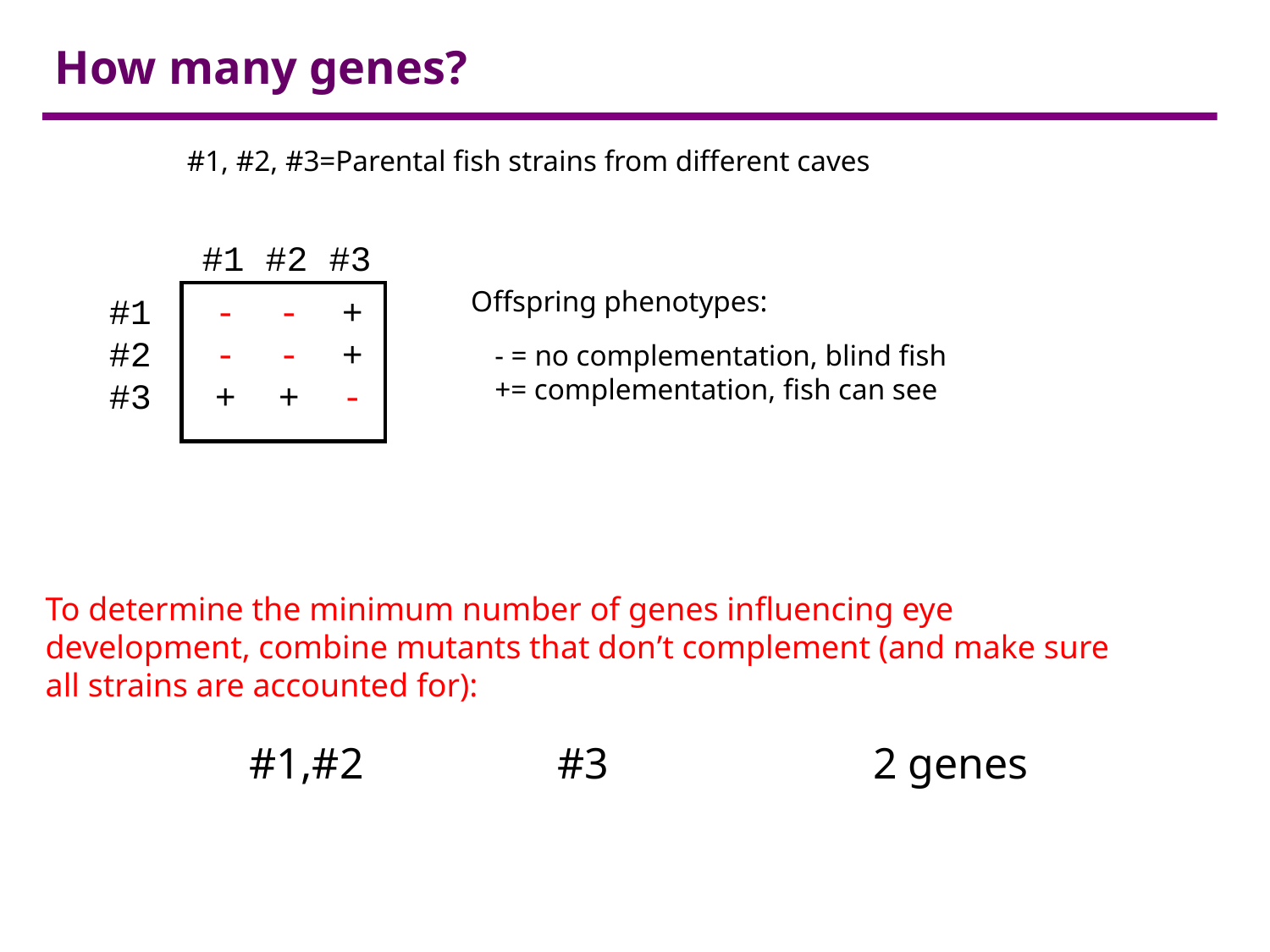

How many genes?
#1, #2, #3=Parental fish strains from different caves
#1	#2 #3
Offspring phenotypes:
- = no complementation, blind fish
+= complementation, fish can see
#1 - - +
#2 - - +
#3 + + -
To determine the minimum number of genes influencing eye development, combine mutants that don’t complement (and make sure all strains are accounted for):
#1,#2
#3
2 genes

## Slide 16
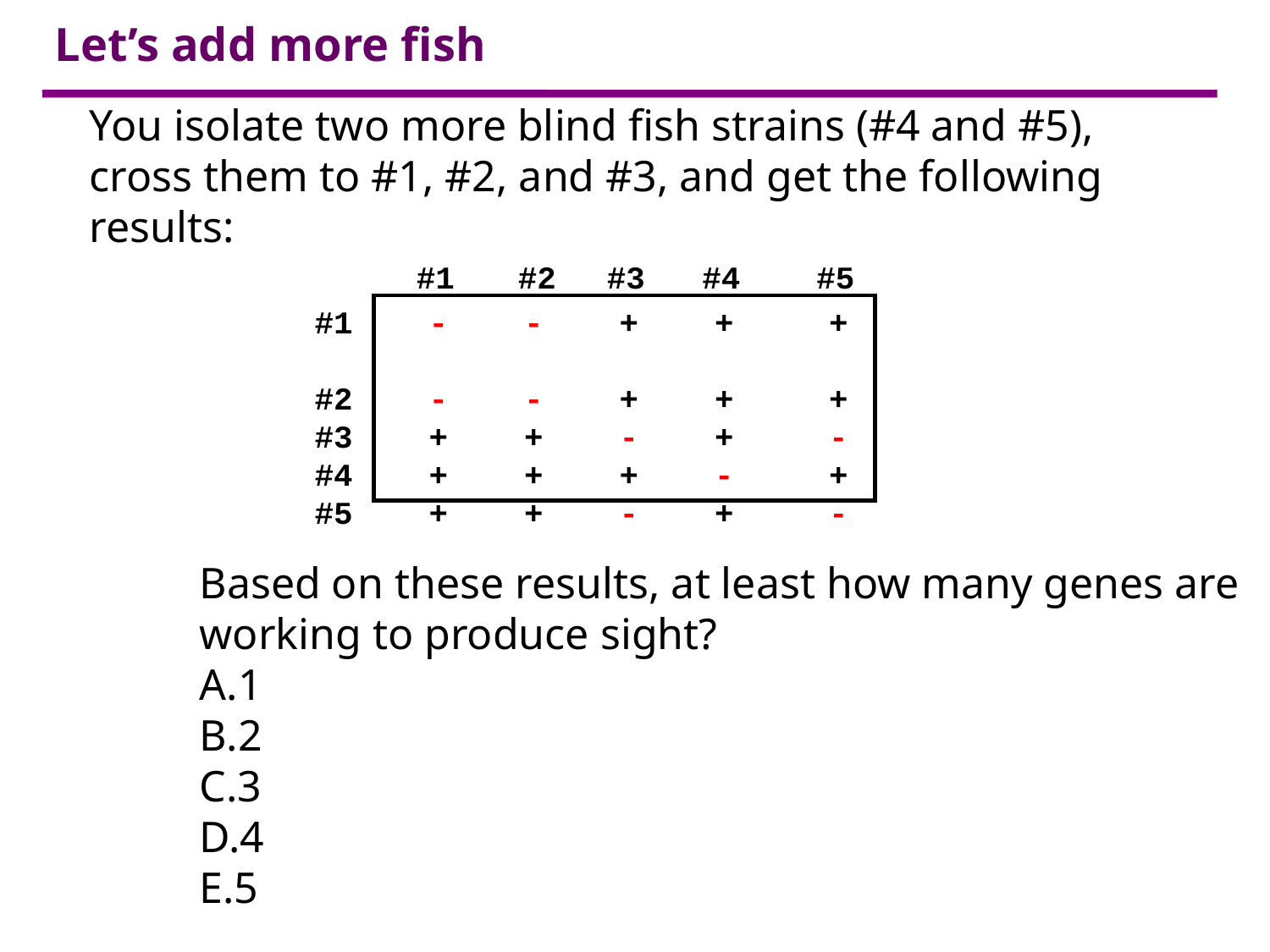

Let’s add more fish
You isolate two more blind fish strains (#4 and #5), cross them to #1, #2, and #3, and get the following results:
#1	 #2	#3 #4 #5
#1 - - + 	 + +
#2 - - +	 + +
#3 + + -	 + -
#4 + + + - +
#5 + + - + -
Based on these results, at least how many genes are working to produce sight?
1
2
3
4
5

## Slide 17
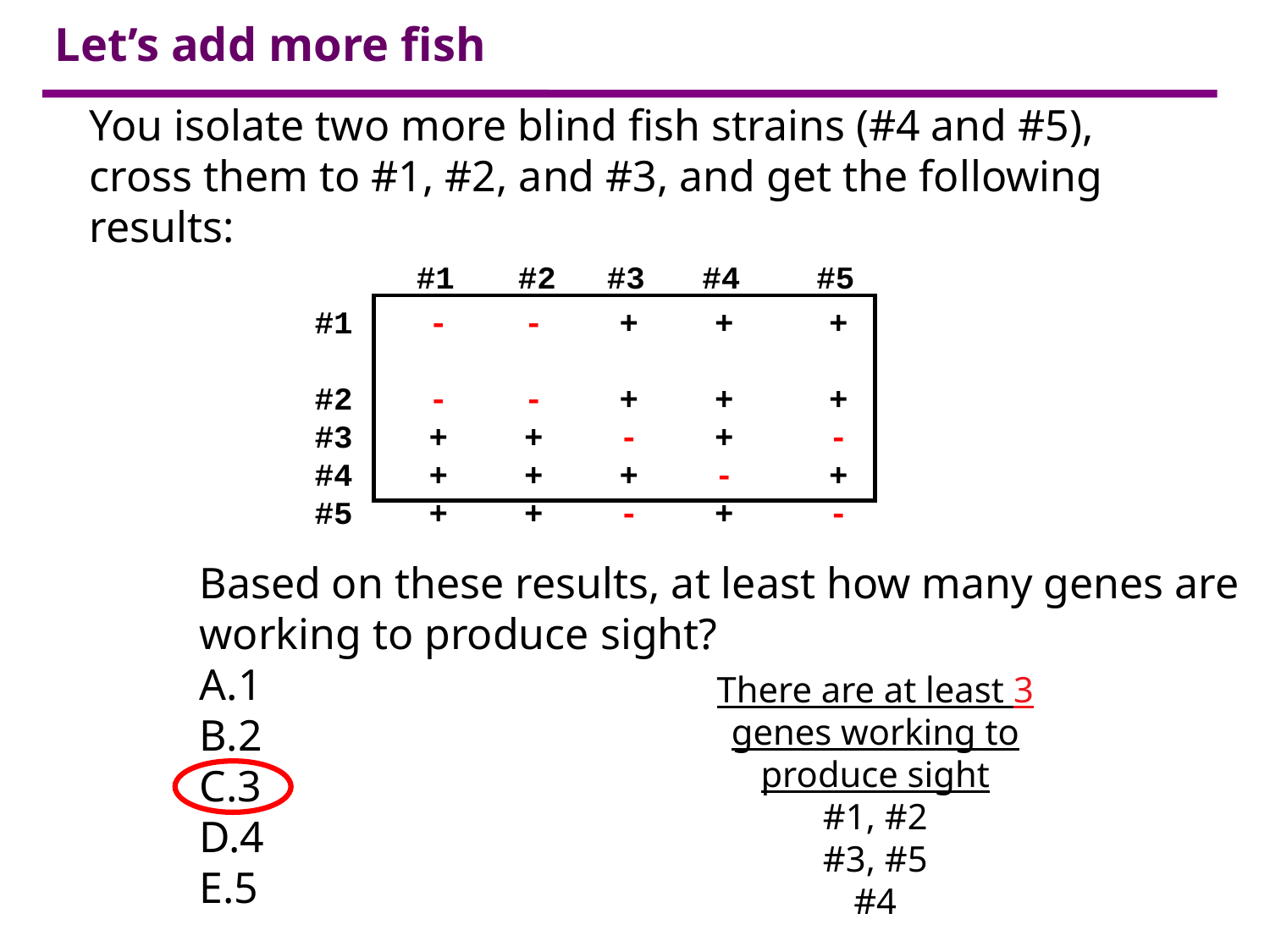

Let’s add more fish
You isolate two more blind fish strains (#4 and #5), cross them to #1, #2, and #3, and get the following results:
#1	 #2	#3 #4 #5
#1 - - + 	 + +
#2 - - +	 + +
#3 + + -	 + -
#4 + + + - +
#5 + + - + -
Based on these results, at least how many genes are working to produce sight?
1
2
3
4
5
There are at least 3 genes working to produce sight
#1, #2
#3, #5
#4

## Slide 18
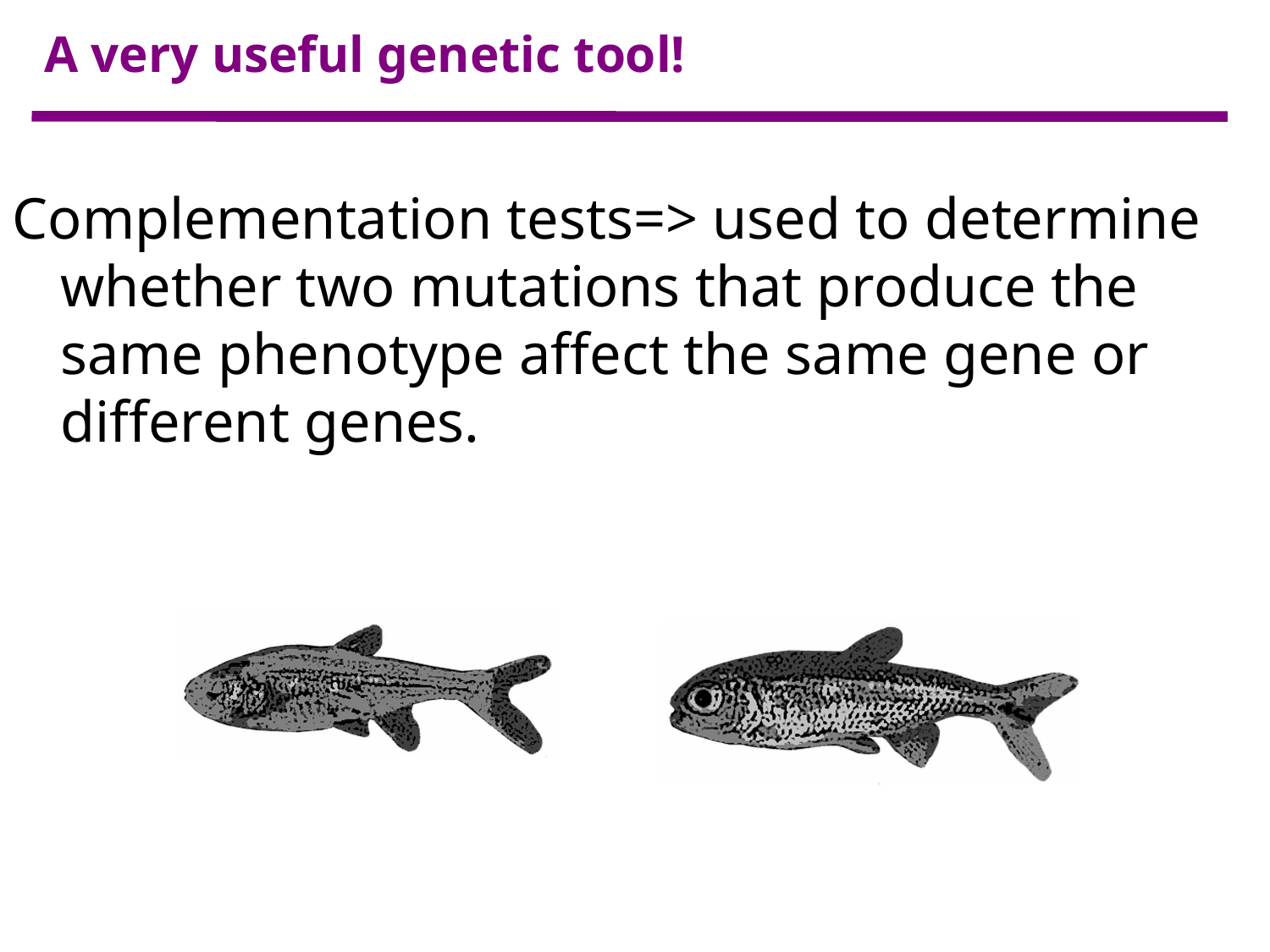

A very useful genetic tool!
Complementation tests=> used to determine whether two mutations that produce the same phenotype affect the same gene or different genes.

## Slide 19
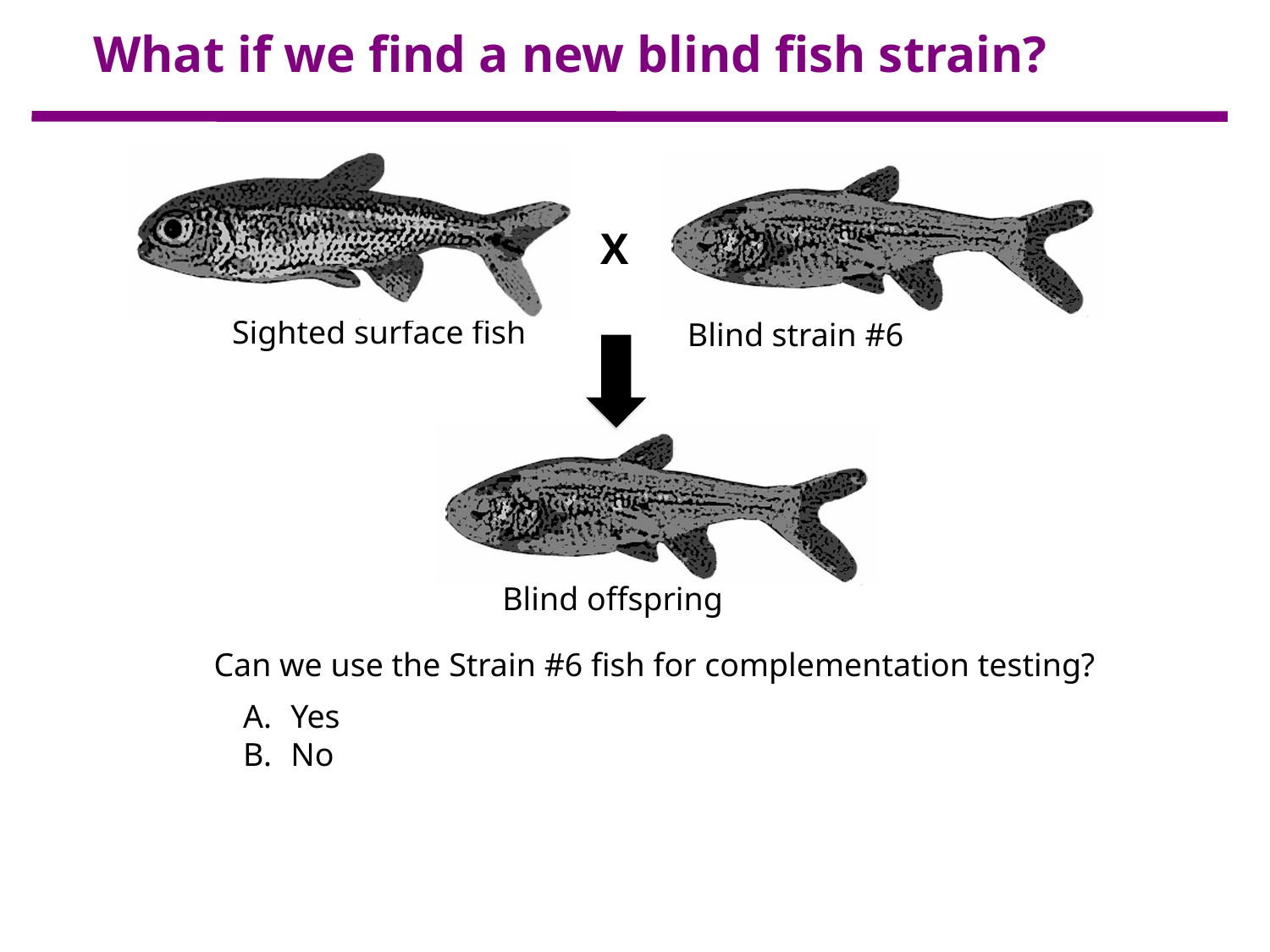

What if we find a new blind fish strain?
X
Sighted surface fish
Blind strain #6
Blind offspring
Can we use the Strain #6 fish for complementation testing?
Yes
No

## Slide 20
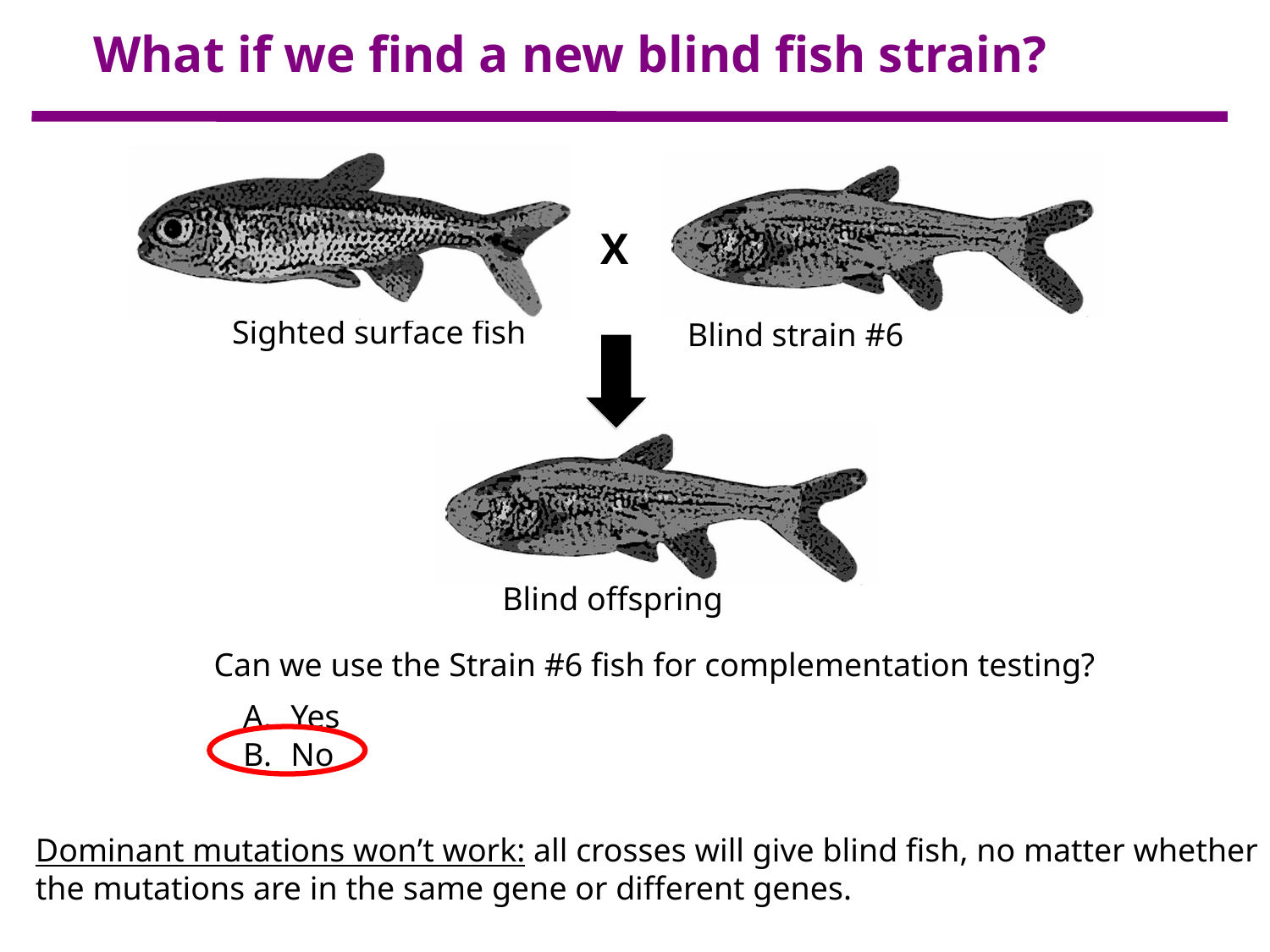

What if we find a new blind fish strain?
X
Sighted surface fish
Blind strain #6
Blind offspring
Can we use the Strain #6 fish for complementation testing?
Yes
No
Dominant mutations won’t work: all crosses will give blind fish, no matter whether the mutations are in the same gene or different genes.
